# Metabolic memory underlying minimal residual disease in breast cancer

**DOI:** 10.15252/msb.202010141

**Published:** 2021-10-25

**Authors:** Ksenija Radic Shechter, Eleni Kafkia, Katharina Zirngibl, Sylwia Gawrzak, Ashna Alladin, Daniel Machado, Christian Lüchtenborg, Daniel C Sévin, Britta Brügger, Kiran R Patil, Martin Jechlinger

**Affiliations:** ^1^ European Molecular Biology Laboratory (EMBL) Heidelberg Germany; ^2^ The Medical Research Council Toxicology Unit University of Cambridge Cambridge UK; ^3^ Biochemie‐Zentrum der Universität Heidelberg (BZH) Heidelberg Germany; ^4^ Cellzome GmbH Functional Genomics GlaxoSmithKline Heidelberg Germany; ^5^ MOLIT Institute gGmbH Heilbronn Germany; ^6^ Present address: Cellzome GmbH Functional Genomics GlaxoSmithKline Heidelberg Germany; ^7^ Present address: Norwegian University of Science and Technology Trondheim Norway

**Keywords:** glycolysis, metabolic modeling, multi‐omics integration, oncogenic memory, organoids, Cancer, Metabolism

## Abstract

Tumor relapse from treatment‐resistant cells (minimal residual disease, MRD) underlies most breast cancer‐related deaths. Yet, the molecular characteristics defining their malignancy have largely remained elusive. Here, we integrated multi‐omics data from a tractable organoid system with a metabolic modeling approach to uncover the metabolic and regulatory idiosyncrasies of the MRD. We find that the resistant cells, despite their non‐proliferative phenotype and the absence of oncogenic signaling, feature increased glycolysis and activity of certain urea cycle enzyme reminiscent of the tumor. This metabolic distinctiveness was also evident in a mouse model and in transcriptomic data from patients following neo‐adjuvant therapy. We further identified a marked similarity in DNA methylation profiles between tumor and residual cells. Taken together, our data reveal a metabolic and epigenetic memory of the treatment‐resistant cells. We further demonstrate that the memorized elevated glycolysis in MRD is crucial for their survival and can be targeted using a small‐molecule inhibitor without impacting normal cells. The metabolic aberrances of MRD thus offer new therapeutic opportunities for post‐treatment care to prevent breast tumor recurrence.

## Graphical abstract

Despite their normal morphology and non‐proliferative phenotype, treatment‐resistant breast cancer cells retain epigenetic imprinting and metabolic characteristics of their prior tumour state.

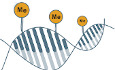

## Introduction

Recent advances in drug development and targeted therapy successfully manage to silence the tumor driving oncogenes, leading to tumor regression and treatment success (Varmus *et al*, 2005; Weinstein & Joe, 2008). However, survival of treatment‐resistant cancer cells, which are commonly referred to as minimal residual disease (MRD), poses a severe problem. MRD is estimated to lead to mostly incurable relapse in 20–40% of breast cancer patients within a period from a few years up to decades after the initial treatment (Aguirre‐Ghiso, 2007; Ahmad, 2013). Understanding and tackling MRD is therefore a major challenge to improving treatment options for breast cancer survivors (Klein, 2011; Cancer Research UK, 2017).

Minimal residual disease is not accessible for direct functional analysis in breast cancer patients due to the small number of these cells and the lack of yet any clear markers to identify them within the patient tissue (Blatter & Rottenberg, 2015). To access the otherwise difficult‐to‐obtain residual cells, a preclinical mouse model of breast cancer was previously developed, together with a primary mammary organoid culture obtained from these mice (Moody *et al*, 2002; Podsypanina *et al*, 2008; Jechlinger *et al*, 2009; Jechlinger, 2015; Havas *et al*, 2017). These models harbor doxycycline‐inducible oncogenes encoding hMyc and Neu/Her2, which allow controlled tumor induction and regression of tumors toward MRD. The resulting tractable models were already successfully employed to compare treatment‐resistant cells to normal breast cells and discovered a de‐regulated lipid metabolism together with oxidative stress as a potential Achilles heel for MRD (Havas *et al*, 2017). Here, we build on these results to obtain a detailed molecular understanding of MRD through a comprehensive comparison of the normal, tumor, and treatment‐resistant cell state. We developed assays for 3D cultures that enabled us to define the molecular states of these three cell populations at epigenetic, transcriptomic, and metabolic levels. The 3‐way comparison between the normal, tumor, and resistant cells, together with genome‐scale metabolic flux modeling, provided a holistic view of the metabolic pathophysiology underpinning the malignancy of the MRD.

## Results

### MRD and the tumor state share characteristic metabolic features

**Figure 1:**
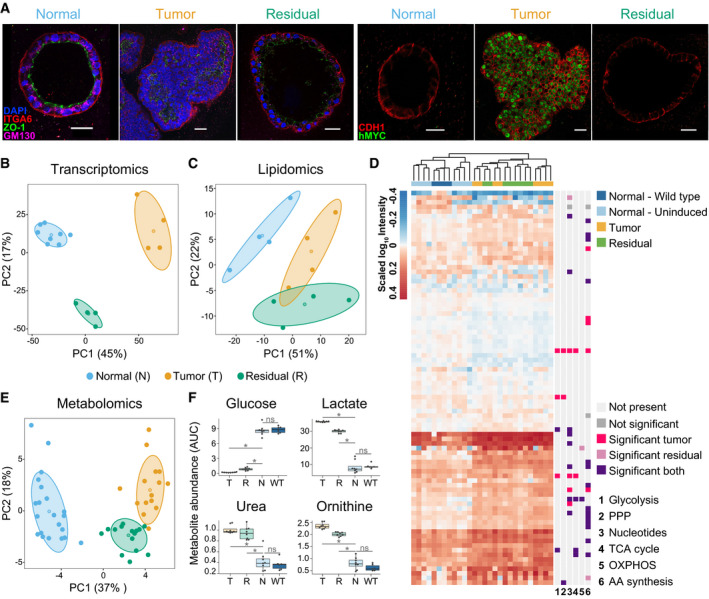
A multi‐omics approach reveals characteristic features of residual organoids *in vitro* and their metabolic resemblance to tumor organoids ARepresentative immunofluorescence staining of normal, tumor, and residual 3D organoids grown from primary mammary cells of a preclinical mouse model of breast cancer (Materials and Methods). Similar morphology of normal and residual organoids shown on the left with polarity markers ITGA6 (red), ZO‐1 (green), GM‐130 (magenta); DAPI (blue). Right: human MYC oncoprotein (green) is expressed only in tumor cells, CDH1 (red). Scale bar: 25 μm.B, CPrincipal component (PC) analyses of normal (blue), tumor (orange), and residual (green) cells based on (B) RNA sequencing data (normal, *n* = 8; tumor and residual, *n* = 4 each) and (C) lipidomic data (*n* = 4).DHeat map of untargeted metabolomics results showing the clustering of the normal—wild‐type (dark blue), normal—uninduced (light blue), tumor (yellow), and residual (green) populations and the most altered metabolic pathways (*n* = 4). Hierarchical clustering was based on the complete linkage method using the Euclidean distance metric. Significance is defined as a false discovery rate < 0.05 vs the normal population, calculated using unpaired two‐sided *t*‐tests and adjusted for multiple hypothesis testing according to Storey's and Tibshirani's method (Storey & Tibshirani, 2003) (Materials and Methods). PPP, pentose phosphate pathway; TCA, tricarboxylic acid cycle; OXPHOS, oxidative phosphorylation; AA, amino acids.EPrincipal component analysis of normal and WT (blue), tumor (orange), and residual (green) populations based on intracellular metabolomics targeted at central carbon metabolism (*n* = 8 each; WT *n* = 2).FSelection of the most profoundly altered metabolites in extracellular spent growth medium from normal (normal and WT, *n* = 4 and *n* = 2, respectively), tumor (*n* = 4), and residual (*n* = 4) populations, based on metabolomics analysis targeted at central carbon metabolites. Values represent metabolite abundance levels as quantified by the area under the curve (AUC) of the marker fragment ions/transitions for each metabolite. Statistics were calculated using the limma package in R (Ritchie *et al*, 2015). Significant results (marked with *) correspond to a Benjamini–Hochberg‐adjusted *P*‐value ≤ 0.05 (residual or tumor compared to normal). ns, not significant. Box plots: midline, median; box, 25–75^th^ percentile; whisker, minimum to maximum. Data information: (A–F), Number of replicates corresponds to individual mice used to derive organoids. (B, C, E) Centroids represent the mean, and concentration ellipses represent one standard deviation (level = 0.68) of an estimated *t*‐distribution based on the first two principal components.

We started with polarized organoids lining a lumen (Fig 1A, referred to as “normal cells” henceforth) that represent the healthy tissue (Jechlinger *et al*, 2009; Havas *et al*, 2017). The addition of 200 ng/ml doxycycline (Appendix Fig S1A–D) activated oncogene expression (Fig 1A, right). This expression led to uncontrolled proliferation as well as the loss of polarity and lumen (Fig 1A), yielding tumor organoids (referred to as “tumor cells” henceforth). Removal of doxycycline from the culture medium silenced oncogene expression triggering tumor regression. Residual organoids (referred to as “residual cells” henceforth) exhibited a re‐polarized epithelium and an absence of hMYC protein (Fig 1A). Together with a near absence of proliferative cells that was previously noted (Jechlinger *et al*, 2009), these observations underscore the dormant nature of these residual cells.

Despite the phenotypic similarity of the residual and normal organoids (Fig 1A), RNA sequencing data showed that the residual cells harbor a transcriptional profile distinct from that of the normal cells (Fig 1B, Appendix Fig S2A). Gene set enrichment analysis comparing residual and normal cells revealed enrichment of downregulated genes in categories such as “cell division and cell cycle” and “cell signaling” (Appendix Table S1, Appendix Fig S2B), again reflecting the dormancy of residual cells. Genes connected to “cytoskeletal organization”, “cell adhesion”, and “cell surface receptor signaling and cytokine production” were upregulated (Appendix Table S2, Appendix Fig S2C), supporting the re‐polarization process upon MRD establishment. While the three cell populations—normal, tumor, and residual—were distinct at the overall transcriptomic level (Fig 1B, Appendix Fig S2D), many differentially expressed genes of the residual cells were shared with the tumor cells (Appendix Fig S2A, D and E). Growth‐supporting metabolic pathways, such as glycolysis and the pentose phosphate pathway, were strongly enriched for upregulated genes (Appendix Fig S2C and E), suggesting that treatment‐resistant, residual cells may be metabolically abnormal.

To detail the metabolic states of the three cell populations, we embarked on lipidomic profiling as well as untargeted and targeted metabolomic analyses. Both intracellular and extracellular (culture supernatant) samples were analyzed to obtain a comprehensive overview of metabolic (patho)physiology. Following optimization of the metabolite extractions from 3D cultures for matrix background noise reduction (Appendix Fig S3A–C), we used shotgun lipidomics to attest the close similarity between residual and tumor populations in agreement with our previous work (Fig 1C) (Havas *et al*, 2017). Beyond lipids, this similarity was also evident in the untargeted metabolomic analysis: The residual cells resembled the tumor cells and not the normal cells (Fig 1D). Importantly, control samples obtained from wild‐type mice lacking the reverse tetracycline‐controlled transactivator (rtTA), but treated with doxycycline (Fig 1D, dark blue), clustered with the normal cells (Fig 1D, light blue), thus excluding a potential confounding effect of doxycycline on metabolism.

Metabolomic analysis targeted at central carbon metabolism confirmed the results obtained from lipidomics and untargeted metabolomics (Fig 1E, Appendix Figs S4A and, S5A and B), validating that dormant residual cells retain key characteristics of their past tumor state. Notably, residual cells aligned with tumor cells in terms of a universal cancer metabolic feature: elevated glycolysis. This alignment was reflected in the decreased glucose levels concomitant with increased levels of lactic acid in spent medium from residual cells and tumor cells in comparison with normal cells (Fig 1F, Appendix Fig S5B). Another prominent metabolic change shared by the normal and residual cells was an increase in secreted urea and ornithine levels (Fig 1F, Appendix Fig S5B), suggesting a dysregulation in urea cycle enzymes. These data are consistent with recent studies pointing to an aberrant expression in many tumor types (Lee *et al*, 2018). Among other metabolic changes (Appendix Fig S5A and B), metabolites connecting the central metabolic pathways with the urea cycle, viz., putrescine, proline, fumarate, and aspartate, were also altered in the intracellular and/or extracellular environment of both tumor and residual cells (Appendix Fig S5A and B). Together, the metabolomic data highlighted the marked similarity in metabolic aberrations of the tumor and residual cell populations.

### Integrative genome‐scale metabolic modeling identifies altered metabolic pathway activities

As changes in intracellular metabolite pools do not necessarily reflect flux changes, we next used a novel integrative genome‐scale metabolic modeling approach to combine transcriptomic and extracellular metabolomic data with flux balance analysis. In brief, the method finds metabolic flux changes that are consistent with mass balance constraints as well as in optimal concordance with the changes in gene expression and measured metabolite secretions.

Since proliferation rates are difficult to accurately measure in the organoid system (especially in the case of the residual cells), the modeling was performed by assuming a constant total metabolic flux. While this assumption limits the interpretation at the level of absolute flux changes between the populations, it allows predicting relative shifts in the flux distribution between different pathways.

**Figure 2:**
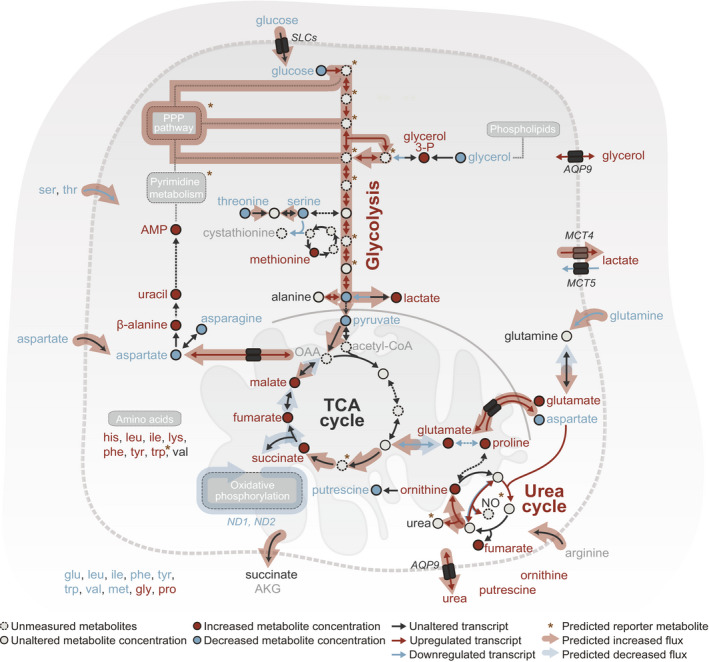
Integrative genome‐scale metabolic modeling identifies altered metabolic pathways in residual cells compared with normal cells A selection of genes with significantly altered expression (Bonferroni‐adjusted *P* < 0.1; normal, *n* = 8; tumor and residual, *n* = 4), targeted metabolites with significantly altered levels (Benjamini–Hochberg‐adjusted *P* ≤ 0.05; normal, tumor, and residual, *n* = 8; WT *n* = 2), and significant reporter metabolites (top 5% with *P* < 0.1) of core metabolic processes are presented. A two‐sided Wald test with a Negative Binomial GLM (Love *et al*, 2014) was used as test statistics for gene expression. For metabolite levels, statistics were calculated using the limma package (Ritchie *et al*, 2015) in R with the significance threshold corresponding to a Benjamini–Hochberg‐adjusted *P*‐value ≤ 0.05 (residual compared to normal). For the reporter metabolites, a gene set enrichment analysis was performed from a theoretical null distribution using the reporter method (Varemo *et al*, 2013). Bonferroni‐adjusted *P*‐values of the expression analysis were used as gene‐level statistics. Metabolite‐gene sets were derived from a genome‐wide human metabolic model (HMR2), with genes mapped to mouse orthologs (Mardinoglu *et al*, 2014). Flux balance analysis predicted metabolic fluxes, which are altered in the corresponding (overlaid) pathways. Significantly altered gene expressions and metabolites were used to inform the predictions. Number of replicates corresponds to individual mice used to derive organoids.

Additionally, we performed a reporter metabolite analysis (Patil & Nielsen, 2005), which uses gene expression changes and metabolic‐network topology to identify de‐regulated metabolites (suggestive of changes in their turnover rate; Materials and Methods). Both genome‐scale flux estimates and the reporter metabolite analysis (Fig 2, Appendix Figs S6 and S7) corroborated our metabolite measurements (Fig 1F, Appendix Fig S5), highlighting the upregulation of glycolysis and urea secretion as major hallmarks of residual and tumor cells. Additionally, supporting the deregulation in parts of urea cycle, an increased aspartate production and arginine uptake was predicted for the residual cells (Fig 2, Appendix Fig S7). The modeling also indicated an increased flux through the pentose phosphate pathway and an increased glutamine uptake in tumor and residual cells, the latter being directed into the TCA cycle in the case of the residual cells (Fig 2, Appendix Figs S6 and S7). The flux through oxidative phosphorylation (OXPHOS) in relation to the glycolysis was predicted to be lower in both residual and tumor cells than in the normal cells. Yet, transcript levels of OXPHOS‐associated genes indicated that the tumor cells harbored higher capacity for OXPHOS than utilized at the flux level (Appendix Fig S8A and B). The excess capacity of tumors in central metabolic pathways has been noted before as a buffer in the face of perturbations (Benard *et al*, 2006; Park *et al*, 2019). Further, the reporter metabolite analysis predicted *S*‐adenosyl methionine (SAM) metabolism‐related alterations for both tumor and residual cells, including DNA and protein methylation as well as DNA replication and repair related metabolites (Appendix Figs S6 and S7).

Together, our transcriptomic data, metabolomic data, and flux modeling revealed marked metabolic peculiarities of the residual cell population. Despite the non‐proliferative status of residual cells, these changes center on growth‐associated metabolic pathways strongly reminiscent of the tumor state (compare Appendix Figs S6 and S7). Residual cells thus appear to carry a “metabolic memory” of the prior tumor state.

### Glycolysis and parts of the urea cycle are also found altered in residual cells in human datasets

**Figure 3:**
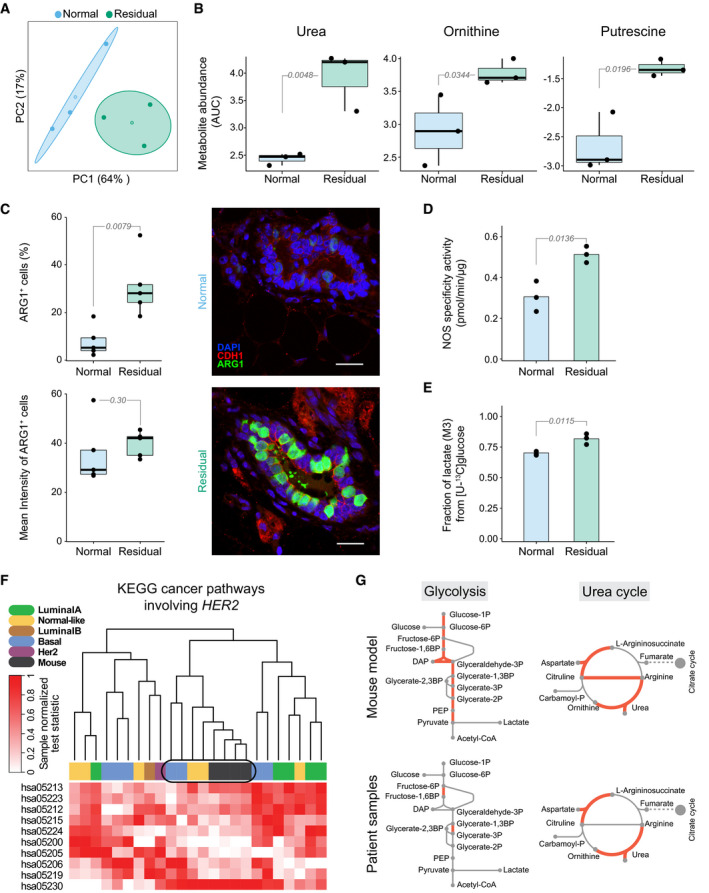
Glycolysis and urea cycle components are the main altered metabolic pathways in residual cells in mice and in human datasets Principal component analysis of extracellular metabolic profiles of isolated healthy (*n* = 3; blue) and regressed (*n* = 3; green) mammary glands after cultivation in cell growth medium for 8 h (Materials and Methods). The metabolomic analysis was targeted to central carbon metabolites. Centroids represent the mean, and concentration ellipses represent one standard deviation (level = 0.68) of an estimated *t*‐distribution based on the first two principal components.Selective secreted metabolites with significant change linked to urea cycle components from healthy (*n* = 3; blue) and regressed (*n* = 3; green) mammary glands. Values represent metabolite abundance levels as quantified by the area under the curve (AUC) of the marker fragment ions/transitions for each metabolite. Values are plotted on the log_2_ scale. Statistics were calculated using the limma package (Ritchie *et al*, 2015) in R with the significance threshold corresponding to a Benjamini–Hochberg‐adjusted *P*‐value ≤ 0.05 (residual compared to normal).Left, Quantification of cells expressing ARG1 (top), an enzyme converting arginine to urea and ornithine, and intensity of ARG1 (bottom) in normal cells from healthy (*n* = 5, 2,921 cells analyzed; blue) and residual cells from regressed (*n* = 5, 2,241 cells analyzed; green) mammary gland tissue sections. Statistical differences were calculated with the Mann–Whitney *U*‐test (Wilcoxon rank‐sum test). Right, Representative images of immunofluorescence staining in normal (top) and residual (bottom) duct stained for ARG1 (green), CDH1 (red), and DAPI (blue). Scale bar: 20 μm.Nitric oxide synthase (NOS) activity, an enzyme involved in arginine metabolism, in healthy (*n* = 3; blue) and in residual (*n* = 3; green) mouse mammary glands. The difference is statistically significant by unpaired two‐sample *t*‐test.Fractional labeling of lactate after cultivation of isolated regressed (*n* = 3; green) and healthy (*n* = 3; blue) mouse mammary glands in cell growth medium supplemented with [U‐^13^C] glucose for 8 h (Materials and Methods). The three‐carbon labeled (^13^C) isotopologue (M + 3) of lactate is depicted and shows enrichment in the residual cells of the regressed mammary glands. The difference is statistically significant by unpaired two‐sample *t*‐test.Joint clustering of sample‐wise normalized pathway enrichment test statistics (unpaired one‐side two‐sample *t*‐test) of mouse model (RNA‐seq; normal, *n* = 8; residual *n* = 4) and patient (microarray; healthy, *n* = 10, regressed *n* = 20). Clustering is based on all genes of human KEGG pathways (or their mouse orthologs) that involve HER2 and are known to be de‐regulated in cancer (Materials and Methods). Hierarchical clustering with the complete linkage method and the Euclidean distance as a distance metric was used for clustering. For the patient comparison, two independent datasets, one from healthy breast tissue (GSE65194) (Maire *et al*, 2013; Maubant, Tesson *et al*, 2015) and one from patient tissues after neo‐adjuvant treatment (GSE32072) (Gonzalez‐Angulo *et al*, 2012), were merged.Metabolic reactions of glycolysis and the urea cycle; differentially expressed (treated patients vs healthy tissue; Benjamini–Hochberg‐adjusted *P* < 0.1) enzymes are highlighted in red. An empirical Bayes moderated *t*‐statistic was computed from a gene‐wise linear model fit with generalized least squares (Ritchie *et al*, 2015), comparing treated patients (*n* = 4 samples), which cluster closely with the mouse samples (encircled in f), with healthy breast tissue (*n* = 10 samples). Differential expression data from mouse *in vitro* transcriptome data of residual vs normal samples (RNA‐seq; normal, *n* = 8; residual, *n* = 4) are shown (two‐sided Wald test (Love *et al*, 2014), Bonferroni‐adjusted *P* < 0.1). Data information: (A–F), Numbers of replicates correspond to individual mice or humans. (B, C) Box plots: midline, median; box, 25–75^th^ percentile; whisker, minimum to maximum. (B–E) Numbers marking comparisons (gray lines) show *P*‐values (corresponding statistical tests are described in individual panel legends).

We next sought to determine the extent to which the metabolic peculiarities of the residual cells in our organoid system capture the situation in mice and in patients. Mouse mammary glands taken nine weeks after oncogene silencing and subsequent tumor regression were compared with healthy glands from age‐matched control animals. Metabolic changes persisted in the residual cells of the regressed tissue (Fig 3A), as reflected in elevated extracellular levels of urea, ornithine, and putrescine (Fig 3B), a higher percentage of arginase 1‐positive cells (Fig 3C, Appendix Fig S9), higher NOS activity (Fig 3D), and higher glycolytic flux to lactate (Fig 3E). To assess the metabolic alterations in patients, transcriptomic datasets of breast tissues after neo‐adjuvant treatment were compared with data from breast tissues from healthy women. Notably, treated patient samples clustered—based on genes in KEGG pathways de‐regulated in cancer and involving *HER2*—closely with the residual mouse samples (Fig 3F) and showed similar alterations in glycolysis and the urea cycle enzymes (Fig 3G). Additionally, transcriptional alterations in these two pathways were evident in the patient samples that were classified as HER2‐positive by histological analysis in the clinic (Appendix Fig S10A), and in samples that clustered closely with the mouse samples based on genes involved in *HER2*‐ or *MYC*‐related KEGG pathways (Appendix Fig S10B and C). These results corroborate the relevance of our 3D culture system and point to a de‐regulated metabolism, in particular glycolysis, in the residual cells in patients.

### Residual cells require elevated glycolysis for survival

Given that glycolysis appeared as a hallmark of MRD in the 3D culture system, in the parental mouse model as well as in the patient data, we hypothesized that targeting this pathway could be a powerful strategy to counter MRD (DeBerardinis *et al*, 2008; Ward & Thompson, 2012). This avenue is particularly attractive because existing FDA‐approved drugs and other approaches targeting tumor metabolism (Ganapathy‐Kanniappan & Geschwind, 2013; Elia *et al*, 2016; Luengo *et al*, 2017; Kanarek *et al*, 2020) could be repurposed against MRD. Our data further suggest that residual cells are likely even more sensitive to these drugs than tumor cells, since residual cell metabolism is overall transcriptionally less perturbed than tumor cell metabolism relative to the normal cellular metabolism (401 differentially expressed metabolic genes in the residual‐normal comparison vs 1,322 in the tumor‐normal comparison; AppendixFig S2F); thus, residual cells are likely to be metabolically less flexible and more reliant on glycolysis than tumor cells. Additionally, our transcriptional data indicate that cells from MRD have a lower OXPHOS capacity than tumor cells further sensitizing them to the loss of glycolysis (Appendix Fig S8A and B). Moreover, residual cells have less anti‐apoptotic signaling pathway activity than tumor cells (Havas *et al*, 2017), suggesting an additional vulnerability of residual cells toward inhibition of key metabolic pathways they over activate and appear to rely on.

**Figure 4:**
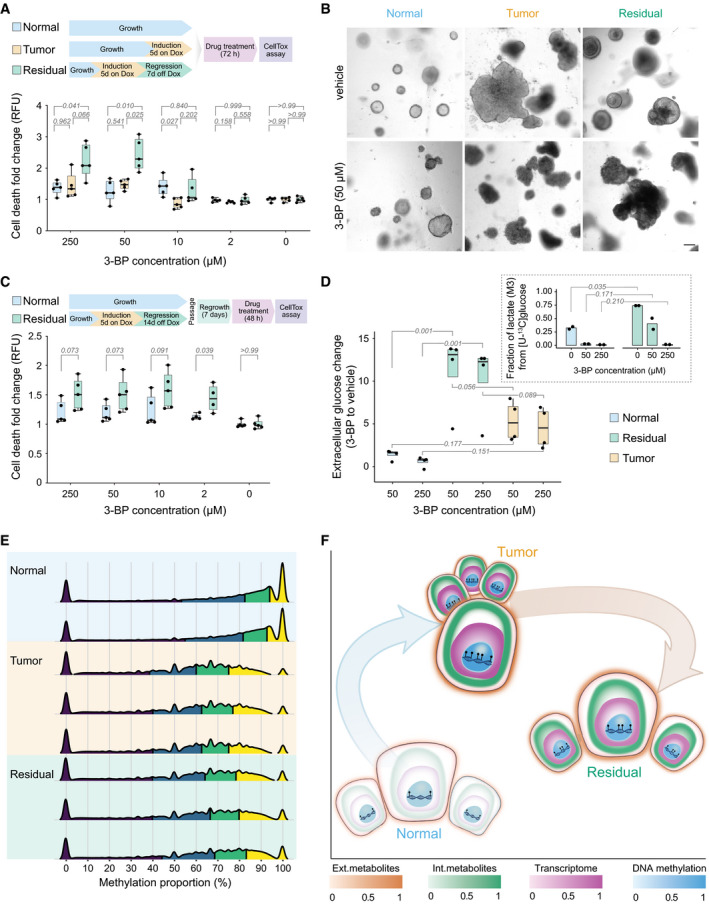
Residual cells require elevated glycolysis for survival and maintain a DNA methylation profile similar to that of tumor cells (Top) Experimental design and (bottom) quantification of cell death (Materials and Methods) of normal, tumor, and residual cells after 72‐h treatment with 3‐BP at the indicated doses (*n* = 5 mice per condition). Two‐way ANOVA with Tukey's HSD multiple comparison testing was utilized to calculate statistical significance. Dox, doxycycline.Representative bright‐field images of normal (left), tumor (middle), and residual (right) organoids, treated with vehicle (top) or with 50 μM 3‐BP (bottom). Scale bar, 100 μm.(Top) Experimental design and (bottom) cell death quantification of passaged residual and normal cells after 48 h of treatment with 3‐BP (*n* = 5 mice per condition). Statistics were calculated with multiple *t*‐tests.(Main) Extracellular glucose abundance changes upon treatment with 3‐BP in all three populations (*n* = 4 mice per condition). Values represent the ratio of glucose abundance in 3‐BP‐treated vs untreated cells. Statistics were calculated using the limma package (Ritchie *et al*, 2015) in R with the significance threshold corresponding to a Benjamini–Hochberg‐adjusted *P*‐value ≤ 0.05 (residual compared to normal). (Insert) Fractional labeling of lactate in untreated and 3‐BP‐treated normal (*n* = 2) and residual (*n* = 2) cells following cultivation in growth medium supplemented with [U‐^13^C] glucose. The three‐carbon labeled (^13^C) isotopologue (M + 3) of lactate is depicted. Statistics were calculated with unpaired two‐sample *t*‐tests.DNA methylation profiles (measured by bisulfide sequencing) of normal (*n* = 2 mice), tumor (*n* = 3 mice), and residual (*n* = 3 mice) cell structures, showing a striking similarity between residual and tumor cells. Plotted are all quantified CpG sides with more than five reads per condition, randomly down‐sampled to 10,000,000 sides. Colors in the density plots represent quartiles.Summary figure integrating transcriptomics (normal, *n* = 8 mice; tumor and residual, *n* = 4 mice each), intracellular (Int.) metabolomics (normal, tumor, and residual, *n* = 8 mice each; WT, *n* = 2 mice), extracellular (Ext.) metabolomics of spent growth medium (normal, *n* = 4 mice; WT, *n* = 2 mice; tumor and residual, *n* = 3 mice each), and DNA methylomics (normal, *n* = 2 mice; tumor and residual, *n* = 3 mice each) from the three populations. Color depth represents the normalized Euclidean distance of the respective omics layer in reference to normal. Distances between the centers of the three populations correspond to the normalized mean Euclidean distances across all represented omics layers. Data information: (A, C, D) Box plots: midline, median; box, 25–75^th^ percentile; whiskers in (A) and (C) minimum to maximum. Number of replicates corresponds to individual mice used to derive organoids. (A, C, D) Numbers marking comparisons (gray lines) show *P*‐values (corresponding statistical tests are described in individual panel legends).

To experimentally test how the inhibition of glycolysis affects normal, tumor, and residual cells, we treated organoids from these three cell populations with 3‐bromopyruvate (3‐BP), a well‐established inhibitor of glycolysis (Fan *et al*, 2019). This treatment induced cell death in residual cells that were cultured with silenced oncogenes for 10 days (Fig 4A, Appendix Fig S11A and B), especially at a dose of 50 µM, which is commonly used for tissue culture (Isayev *et al*, 2014; Chen *et al*, 2018). In contrast, normal and tumor cells stayed close to their baseline viability after exposure to 3‐BP (Fig 4A). The cytotoxic effect of glycolytic inhibition was also reflected in the morphology of the residual organoids, which lost their defined rim and presented as collapsed, dark spheres (Fig 4B, lower panel). At the same time, the normal and the tumor organoids did not appreciably change their appearance compared with the untreated condition (Fig 4B). These results show that residual cells are indeed—as hypothesized above—more dependent on glycolysis than tumor cells at doses of 3‐BP that do not affect normal cells. Increased cell death upon 3‐BP treatment also occurred in residual cells that were passaged to re‐establish secondary organoids, and grew a total of 21 days after oncogene silencing (Fig 4C), reinforcing the notion of a metabolic memory carried over from the tumor state.

Consistent with the cell death assays (Fig 4A and C), quantification of extracellular metabolite pools showed that glucose uptake reduction upon 3‐BP treatment was most dramatic for the residual cells, especially at the 50 and 250 µM doses (Fig 4D). In agreement, we also observed significant alterations in extracellular lactate levels, as well as in other metabolites (e.g., glutamine) in residual cells (Appendix Fig S11B). In contrast, normal cells were affected by 3‐BP to a lesser extent (Fig 4D, Appendix Fig S11B). The higher glycolytic nature of residual cells as compared to normal cells, and the effects of 3‐BP treatment on glycolysis, was further validated by the differential incorporation of glucose‐derived carbons into lactate (Fig 4D). Together, the differences in cell death and metabolite levels attest the glycolytic nature and dependency of the residual cells, indicating glycolysis as a potential therapeutic target.

### Treatment‐resistant cells exhibit DNA methylation profile similar to the tumor cells

Exploring the basis of the metabolic memory of the residual cell population, we observed that several signaling pathways, including the signaling network of hypoxia‐inducible factor 1 (HIF1α), a well‐known master regulator of the glycolytic phenotype in cancers, are distinctively active in residual cells as assessed by a footprinting‐based activity analysis of transcription factor (TF)‐target gene expression (Appendix Fig S12A–C, Appendix Table S3) (Essaghir *et al*, 2010; Alvarez *et al*, 2016; Holland *et al*, 2020). In particular, glycolysis protein isoforms specific for HIF1α signaling, e.g., GLUT1, HK2, PFKL, ALDOA, PGK1, ENO1, PKM, and PFKFB3, were overexpressed (Marin‐Hernandez *et al*, 2009). Further, some of the metabolites that were more abundant in the residual cell populations compared with the normal—such as succinate and lactate (Appendix Fig S5A, Fig 3D)—are known signaling molecules that interface with hypoxic signaling by inducing HIF1α stabilization even under normoxic conditions (Selak *et al*, 2005; King *et al*, 2006; Tannahill *et al*, 2013; Lee *et al*, 2015). Additionally, succinate and nitric oxide as well as other metabolites such as fumarate, all of which are accumulated in residual cells (Appendix Fig S5A, Fig 3D), are also implicated in epigenetic modifications through direct inhibition of alpha‐ketoglutarate‐dependent demethylases (Xiao *et al*, 2012; Vasudevan *et al*, 2015; Miranda‐Goncalves *et al*, 2018). Concurrent with these metabolic changes, we found that the DNA methylation profiles of residual cells closely resembled that of the tumor cells (Fig 4E, Appendix Fig S4B), thus indicating the mechanistic basis of the metabolic memory of the residual population. Promoter regions of HIF1α and glycolytic co‐activator proteins as well as glycolytic target genes of HIF1α are consistently demethylated in tumor and residual cells with isoform‐specific demethylations in both populations (Appendix Fig S13A and B). Together, the persistence of a tumor‐associated metabolic signature in the residual population despite the absence of continued oncogenic input suggests that the accumulation of certain metabolites affords an additional survival advantage for MRD that is sustained through epigenetic imprinting.

## Discussion

Our study offers a first in‐depth characterization of MRD derived from a primary organoid system that is relevant to human disease. We conducted a comprehensive multi‐omics analysis including transcriptomics, untargeted metabolomics, extra‐ and intracellular metabolomics, lipidomics, and methylomics. A novel metabolic model‐based data integration method allowed us to integrate different omics layers, from the transcriptome to the metabolome, and thereby gain insights into flux reorganization underlying the MRD. The method overcomes the limitation of qualitative predictions from logical network methods typically applied in this field and instead allows quantitative assessment of flux (re‐)distributions. This integrative modeling approach is broadly applicable to other systems where metabolic flux changes are central to the disease or other perturbations.

While previous studies have identified some of the metabolic characteristics of residual cells—such as elevated ROS levels—in comparison with tumor material (Viale *et al*, 2014; Havas *et al*, 2017; Fox *et al*, 2020), the three‐way multi‐omics comparison in this study enabled pinpointing the distinctness of residual cells to both normal cells and tumor cells in a holistic fashion. Although residual cells are characterized by a dormant and histologically normal phenotype, they harbor metabolic aberrations that are similar to those of the tumor cells (Fig 4F). We refer to these changes as “metabolic memory of the prior tumor state” or “metabolic memory”, which is reflected in gene transcription, metabolite levels, glycolytic flux, and DNA methylation. We observe that this memory persists without active oncogenic signal in residual cells. Since residual cells constitute a treatment‐resistant population of dormant cancer cells, these memorized aberrations offer a unique therapeutic opportunity.

Our *in vitro* findings based on organoids were validated *in vivo* through analyzing residual mammary glands taken from the mouse model (Fig 3A–E). In addition, data from neo‐adjuvant‐treated patients with breast cancer (Fig 3F and G, Appendix Fig S10A–C) support these conclusions and link the findings to the patient situation. Interference with one of the commonly de‐regulated metabolic nodes in tumor and residual cells, glycolysis, enabled selective targeting of residual cells (Fig 4A–C). The identified transcriptional, metabolic, and epigenetic distinctiveness (Fig 4F) of MRD thus offers targeted treatment options to counter progression toward tumor relapse.

## Materials and Methods

### Reagents and Tools table

**Table:** 

Reagent/resource	Reference or source	Identifier or catalog number
**Experimental Models**		
FVB (*Mus musculus*) GEMM *TetO‐cMYC*/*TetO‐Neu*/*MMTV‐rtTA*	D'Cruz *et al* (2001), Gunther *et al* (2002), Moody *et al* (2002)	N/A
**Antibodies**		
Rat anti‐integrin alpha‐6	Millipore	Cat # MAB1378
Rabbit anti‐ZO‐1	Invitrogen	Cat # 61‐7300
Mouse anti‐GM‐130	BD Biosciences	Cat # 610823
Rat anti‐Cadherin‐1	Invitrogen	Cat # 131900
Rabbit anti‐AR‐G1	Novus Biologicals	Cat # NBP1‐32731
Mouse anti‐MT‐CO1	Abcam	Cat # ab14705
Goat anti‐rat IgG (H + L) Alexa Fluor 647	Invitrogen	Cat # A‐21247
Goat anti‐rabbit IgG (H + L) Alexa Fluor 488	Invitrogen	Cat # A‐11034
Goat anti‐mouse IgG (H + L) Alexa Fluor 568	Invitrogen	Cat # A‐11031
**Oligonucleotides and other sequence‐based reagents**		
TetO‐MYC	https://www.jax.org/Protocol/UrlAsPDF?stockNumber=019376&protocolID=24553	Table EV1
TetO‐Neu	Moody *et al* (2002)	Table EV1
MMTV‐rtTA	Gunther *et al* (2002)	Table EV1
**Chemicals, Enzymes, and other reagents**		
Agarose	Sigma‐Aldrich	A9539
Ethidium bromide	Sigma‐Aldrich	E1510
DMEM:F12 with HEPES, Glucose and l‐Glutamine	Lonza	Cat # BE12‐719F
Collagenase Type 3	Worthington Biochemical	Cat # LS004183
LiberaseTM Research Grade	Roche	05401127001
Pen Strep	Gibco	15140‐122
Trypsin‐EDTA (0.25%)	Gibco	Cat # 25200056
Fetal Bovine Serum (FBS), Tetracycline Free	Biowest	S181T
Deoxyribonuclease I from bovine pancreas	Sigma‐Aldrich	D4527
MEBM Mammary Epithelial Cell Growth Basal Medium	Lonza	Cat # CC‐3151
MEGM Mammary Epithelial Cell Growth Medium BulletKit	Lonza	Cat # CC‐3150
Matrigel Matrix	Corning	354230
3‐D Culture Matrix Rat Collagen I	Cultrex	Cat # 3447‐020‐01
Doxycycline hyclate	Sigma‐Aldrich	D9891
Paraformaldehyde	Sigma‐Aldrich	158127
Sodium phosphate dibasic heptahydrate	Sigma‐Aldrich	S9390
Sodium dihydrogen phosphate monohydrate, Monosodium phosphate	Sigma‐Aldrich	S9638
Sodium azide	Sigma‐Aldrich	S8032
Bovine Serum Albumin	Sigma‐Aldrich	A9418
Triton X‐100	Sigma‐Aldrich	T8787
TWEEN 20	Sigma‐Aldrich	P9416
Normal Goat Serum	Jackson ImmunoResearch	005‐000‐121
VECTASHIELD Hard Set with DAPI	Vector Laboratories	Cat # H‐1500
DAPI Solution	Thermo Scientific	Cat # 62248
ProLong Gold Antifade Mountant	Invitrogen	Cat # P36930
Xylenes	Sigma‐Aldrich	1.08633
Antigen Unmasking Solution	Vector Laboratories	H‐3300
Hydrogen Peroxide Solution	Sigma‐Aldrich	H1009
VECTASTAIN Elite ABC‐HRP Kit, Peroxidase (Mouse IgG)	Vector Laboratories	PK‐6102
DAB Substrate Kit, Peroxidase (HRP)	Vector Laboratories	SK‐1400
Hematoxylin QS Counterstain	Vector Laboratories	H‐3404‐100
DPX Slide Mounting Medium	Sigma	06522
mirVana miRNA Isolation Kit, with phenol	Ambion	Cat # AM1560
HPLC‐grade methanol	Biosolve Chimie	Cat # 136841
Adonitol	Alfa Aesar	488‐81‐3
Crystal Violet solution	Sigma‐Aldrich	V5265
DMEM, no glucose	Gibco	Cat # 11966025
d‐glucose (U‐13C6, 99%)	Cambridge Isotope Lab.	CLM‐1396
DMEM, high glucose, GlutaMAX Supplement	Gibco	Cat # 10569044
Methoxyamine hydrochloride	Alfa Aesar	A19188
Pyridine	Sigma‐Aldrich	437611
*N*‐methyl‐trimethylsilyl‐trifluoroacetamide	Alfa Aesar	A13141
*N*‐tert‐Butyldimethylsilyl‐*N*‐methyltrifluoroacetamide + 1% tert‐Butyldimethylchlorosilane	Sigma‐Aldrich	00942
Nitric Oxide Synthase Activity Assay kit	Abcam	ab211083
**Software**		
Quantum‐Capt1	Vilber Lourmat	
LAS AF v.2.7.3.9723	Leica Microsystems	
StrataQuest v.5	TissueGnostics	
LAS 3.7	Leica Microsystems	
R v3.3.1	R‐Core‐Team (2016)	
Leica Application Suite X	Leica Microsystems	
GCMS Solution	Shimadzu	
R v3.5.2	R‐Core‐Team (2016)	
Isotope Correction Toolbox	Jungreuthmayer *et al* (2016)	
LipidView	Sciex	
ShinyLipids	BZH, Heidelberg University	
Fiji/ImageJ	https://imagej.net/software/fiji/	
GraphPad Prism Version8	https://www.graphpad.com/data‐analysis‐resource‐center/	
**Other**		
Food pellets with Doxycycline hyclate (625 mg/kg)	Envigo Teklad	N/A
Polypropylene conical tubes (50 and 15 ml)	Falcon	352070, 352095
BioCoat Collagen I 6‐well Clear Flat Bottom TC‐treated Multiwell Plate, with Lid	Corning	356400
CellBIND 12‐well Multiple Well Plates, Flat Bottom, with Lid, Sterile	Corning	3336
Nunc Lab‐Tek II Chambered Coverglass	Thermo Scientific	155379
6‐well Black/Clear Flat Bottom TC‐treated Imaging Microplate with Lid	Falcon	353219
Deactivated Clear Glass 12 × 32 mm Screw Neck Vial	Waters	186000989DV
Leica TCS SP5 microscope	Leica Microsystems	
TissueFAXS SL	TissueGnostics	
LMD 7000 microscope	Leica Microsystems	
DFC310FX digital color cam.	Leica Microsystems	
2100 Bioanalyzer Instrument	Agilent	
TruSeq RNA Library Prep Kit	Illumina	
Biomek FX Automated Workstation	Beckman Coulter	
Illumina HiSeq 4000	Illumina	
Illumina NextSeq 500	Illumina	
Genevac EZ‐2 Plus	SP Scientific	
Leica DFC7000T microscope	Leica Microsystems	
GCMS‐TQ8040	Shimadzu	
Zebron™ ZB‐50, GC Cap. Column 30 m × 0.25 mm × 0.25 µm	Phenomenex	
Ultimate 3000 LC coupled to Q‐Exactive Plus MS	Thermo Scientific	
QTRAP 6500+ MS	Sciex	
HD‐D ESI Chip	Advion Biosciences	
Triversa Nanomate	Advion Biosciences	

### Methods and Protocols

#### Animals

Breeding and maintenance of the mouse colony was done in the Laboratory Animal Resources (LAR) facility of EMBL Heidelberg in accordance with the guidelines of the European Commission, revised Directive 2010/63/EU and AVMA Guidelines 2007, under veterinarian supervision. The EMBL Institutional Animal Care and Use Committee (IACUC) approved the work with these mice (approval # MJ160070). The animals—*TetO‐cMYC*/*TetO‐Neu*/*MMTV‐rtTA* (D'Cruz *et al*, 2001; Gunther *et al*, 2002; Moody *et al*, 2002) in FVB background—were kept on a 12‐h light/12‐h dark cycle with a constant ambient temperature (23 ± 1°C) and humidity (60 ± 8%), supplied with food pellets (for tumor induction the pellets contained doxycycline hyclate, 625 mg/kg; Envigo Teklad) and water *ad libitum*.

#### Genotyping

For genotyping, genomic DNA was extracted by tail‐digestion in 75 µl of digestion buffer (NaOH 25 mM + EDTA 0.2 mM) at 98°C followed by the addition of 75 µl Tris–HCl (40 mM, pH 5.5) and centrifugation at 1,500 *g* for 3 min. The sequences of the used primers can be found in Table EV1. Gel electrophoresis was used for the detection of PCR products (*MYC* 630 bp, Neu 386 bp, rtTA 380 bp) on a 1.5% agarose (Sigma, A9539‐500G) gel with ethidium bromide solution (Sigma, E1510‐10ML) in a final concentration of 0.5 µg/ml. The products were visualized using the Quantum‐Capt1 documentation system (Vilber).

#### 3D cell culture

Primary mammary epithelial cells were obtained from 8‐week‐old virgin females of GEMM *TetO‐cMYC*/*TetO‐Neu*/*MMTV‐rtTA* mouse strains in FVB background. Three‐dimensional (3D) cell cultures were established according to the published protocol (Jechlinger *et al*, 2009; Jechlinger, 2015) with some modifications.

1. Reagents that need to be already prepared:
  ‐ Digestion media: 500 ml of Dulbecco's modified Eagle medium:F12, DMEM:F12 with HEPES (15 mM), 1:1 mixture with 3.151 g/l glucose, with l‐glutamine (Lonza BE12‐719F) supplemented with HEPES to the final concentration of 25 mM and 5 ml of penicillin/streptomycin (Gibco, 15140‐122).
  ‐ Collagenase type 3 (Worthington, LS004183) to a working concentration of 75,000 U/ml in digestion media.
  ‐ Liberase (Roche, 05401127001) to a working concentration of 10 mg/ml in digestion media; ‐DNaseI (Sigma, D4527) to a working concentration of 1 mg/ml in digestion media.
  ‐ STOP media: 500 ml of Dulbecco's modified Eagle medium:F12, DMEM:F12 with HEPES (15 mM), 1:1 mixture with 3.151 g/l glucose, with l‐glutamine (Lonza BE12‐719F), supplemented with HEPES to the final concentration of 25 mM, 5 ml of penicillin/streptomycin (Gibco, 15140‐122) and 50 ml of fetal bovine serum, tetracycline free (Biowest, S181T).
  ‐ 500 ml MEBM mammary epithelial cell growth basal medium (Lonza, CC‐3151) supplemented with 2 ml of bovine pituitary extract, 0.5 ml of hEGF, 0.5 ml of hydrocortisone, 0.5 ml of GA‐1000, 0.5 ml insulin from MEGM mammary epithelial cell growth medium BulletKit (Lonza CC‐3150).
    *Warm all the described media, PBS, trypsin‐EDTA to 37°C before using.
2. Prepare labeled 50‐ml polypropylene conical tube (Falcon, 352070) for each pair of harvested mammary glands and mix 5 ml of digestion media (described in Step 1) with 10 µl collagenase and 10 µl liberase into each tube. Warm to 37°C.
3. Harvest mouse mammary glands, transfer to cell culture, and place them into prepared, warm tubes (described in Step 2). Digest the glands for 15–16 h at 37°C in 5% (vol/vol) CO_2_ atmosphere in loosely capped 50‐ml polypropylene conical tubes.
4. After 15–16 h, gently disrupt the digested glands by pipetting and wash them with 45 ml of phosphate‐buffered saline (PBS). Centrifuge at room temperature, 1,000 r.p.m. for 5 min. Remove the interphase between the upper fat layer and the cell pellet (leaving around 5 ml of liquid) and add 5 ml of 0.25% Trypsin‐EDTA (Gibco, 25200056) to each tube, gently resuspending the pellet. Incubate the suspension for 30–40 min at 37°C, 5% CO_2_ in loosely capped tubes.
5. Wash with 25 ml of STOP medium (described in Step 1) and add 7 µl DNase I (per each tube), incubating for 5 min at room temperature.
6. Centrifuge at room temperature, 1,000 r.p.m. for 5 min and aspirate the supernatant. Resuspend the pellet of dissociated cells in MEBM mammary epithelial cell growth basal medium supplemented with MEGM mammary epithelial cell growth medium BulletKit (2–3 ml per tube, combining cells from all tubes per animal and rinsing with MEBM media to obtain maximum yield). Plate 3 ml per well onto collagen‐coated 6‐well plates (Corning, 356400) for the selection of epithelial cells. Leave the plate for ca. 24 h 37°C, 5% CO_2_.
7. On the next day, wash the cells with PBS (each well with 2–3 ml). Remove PBS and treat the remaining cells with 500 µl of 0.25% trypsin‐EDTA per well (3–5 min, 37°C, 5% CO_2_) until detachment.
8. Inactivate trypsin by adding 2 ml of STOP media (described in Step 1) to each well, mix well, and pool cell suspensions from wells per animal into a 15‐ml tube (Falcon, 352095). Rinse the wells with STOP media and add rinses, making up the volume to 10–12 ml per tube. Centrifuge at room temperature, 1,000 r.p.m. for 5 min. Remove the supernatant as much as possible and resuspend the cell pellets in MEBM (depends on the pellet size, use 100–500 µl). Count the cells in the single cell suspension.
9. Prepare Matrigel‐collagen mixture (for number of gels to be seeded)—Matrigel Matrix (Corning, 354230) and 3D culture Matrix Rat Collagen I (Cultrex, 3447‐020‐01) on ice: mix Matrigel, Collagen I and PBS in the ratio of 4:1:1. Mix the components in the following order: cold PBS, Collagen I and Matrigel, compensating volumes for 10% error due to pipetting and mixing gently by pipetting, avoiding air bubbles.
10. Add the prepared Matrigel (volume of 100 µl) into cell suspension (12,000 primary mouse mammary epithelial cells), mix gently, and dispense into flat bottom wells (CellBIND 12 Well Clear Multiple Well Plates, Corning 3336) or chambered coverglass slides for imaging (Nunc LabTek II Chambered Coverglass, Thermo Fisher Scientific, 155379). Leave the plates in the incubator at 37°C, 5% CO_2_ for 30–40 min, for gels to solidify.
11. Add 1.5 ml of MEBM medium supplemented with BulletKit (described in Step 1).

For experiments based on biochemical assays, a suspension of 500 cells and PBS was mixed with Matrigel in a ratio of 1:4 for 5 µl gels that were seeded in the black TC‐treated imaging 96‐well plates (Falcon, 353219) with clear flat bottom and left to solidify for 15 min at 37°C, followed by the addition of 100 µl of MEBM medium.

Subsequently, the cells were grown for 7 days at 37°C, 5% CO_2_ in a humidified incubator until the 3D organoid culture had established including the formation of polarized acini. The experiments were started by the addition of doxycycline (Doxycycline hyclate, Sigma, D9891) to the cell culture medium in a concentration of 200 ng/ml, initiating the transcription of the oncogenes and rapid cell proliferation.

For subset of experiments based on biochemical assays involving secondary organoids (grown 21 days after oncogene silencing timepoint), passaging of cells was done in the following manner: Gels (volume of 100 µl containing 12,000 cells per gel) were digested by adding 3 µl collagenase and 3 µl liberase to the wells with media, with incubation for 1–1.5 h at 37°C, 5% CO_2_. Disintegrated gels were collected to Falcon tube (wells from same samples were pooled) and washed with PBS, then centrifuged at 1,000 r.p.m. for 5 min. PBS was aspirated, and trypsin/EDTA was added to the cells, with incubation for 5–10 min and pipetting at the end to mechanically break up the structures. Deactivation was done with 10 ml STOP media, with DNase added (described in Step 1). Samples were centrifuged at 1,000 r.p.m. for 5 min and resuspended in PBS for counting and seeding, which was done by the protocol described above, for 5 µl gels.

For the RNA sequencing experiments, tumor samples were harvested 5 days after oncogene induction. Subsequently, doxycycline was removed from the cell culture medium, which resulted in a silencing of the oncogenes followed by tumor regression. Residual samples were harvested 7 days after de‐induction. Non‐induced structures were grown in medium without the addition of doxycycline and sampled in parallel to the above described timepoints. The medium was changed every second day with the exception of the doxycycline‐induced cultures, when it was changed daily between third to fifth day (for tumor organoids) and between the first and third day upon de‐induction (residual organoids).

For metabolic experiments, media was changed daily. Induction and de‐induction timepoints were planned in such a way that metabolites were collected and processed in parallel, at the same time for all three conditions (normal, tumor, and residual).

#### Immunofluorescence

3D culture gels for immunofluorescence staining were washed 2× in PBS and transferred to the IF deactivated clear glass screw neck vials (Waters, 186000989DV).

1. Fix with 4% paraformaldehyde (Sigma‐Aldrich, 158127) for 7–10 min, remove, and subsequently wash three times 10 min with PBS and once in freshly made 1× IF buffer (pH 7.4): containing NaCl, Na_2_HPO_4*_7H_2_O (Sigma‐Aldrich, S9390), NaH_2_PO_4*_H_2_O (Sigma‐Aldrich, S9638), NaN_3_ (Sigma‐Aldrich, S8032), BSA (Sigma‐Aldrich, A9418), Triton X‐100 (Sigma‐Aldrich, T8787), Tween‐20 (Sigma‐Aldrich, P9416).
2. Block for 1.5 h using 1× IF buffer with 10% goat serum (Jackson ImmunoResearch, 005‐000‐121).
3. Dilute primary antibodies in primary block (as described in Step 2) and incubate overnight at 4°C.
4. Wash the next day in 1× IF buffer (described in Step 1) for three times, 15 min each.
5. Incubate with secondary antibodies and 4′, 6′‐diamino‐2‐phenylindole (DAPI) diluted in the primary block (described in Step 2) for 1 h.
6. Wash the gels with 1× IF buffer and 1× PBS for two times, 10 min each.
7. Mount the gels with VECTASHIELD Antifade mounting medium (Vector Laboratories, H‐1500) into LabTek II chamber slide (Nunc LabTek II Chambered Coverglass, Thermo Fisher Scientific, 155379).

Gels were imaged on a Leica SP5 confocal microscope (Leica TCS SP5, Leica Microsystems) using a 63× water lens and Leica Application Suite Advanced Fluorescence imaging software (LAS AF, Leica Microsystems). Fiji (Schindelin *et al*, 2012) was used for image analysis (merged images, projection through the acinus from subsequent 5–6 stacks, adjusted color channels—parameters provided in the Source Data). The following antibodies were used for the 3D cultures: alpha‐6‐integrin (Millipore, MAB1378), diluted 1:80), ZO‐1 (Invitrogen 61‐7300, diluted 1:500), GM‐130 (BD Biosciences, BDB610823, diluted 1:100), and E‐cadherin (Invitrogen, 13‐1900, diluted 1:200). Nuclei were stained with DAPI (Invitrogen, 62248, diluted 1:1,000). Anti‐rabbit, anti‐mouse, and anti‐rat antibodies coupled with Alexa Fluor dyes were purchased from Invitrogen (A‐21247, A‐11034, A‐11031, diluted 1:500).

FFPE tissue sections were stained using the standard protocols for ARG1 (Novus, NBP1‐32731, diluted 1:250) antibody. Sections were mounted using ProLong Gold Antifade Mountant (Invitrogen, P36930), imaged on Leica TCS SP5 (Leica Microsystems) using a 63× water lens, LAS AF (Leica Microsystems) imaging software and analyzed in Fiji (merged images, adjusted color channels—parameters provided in the Source Data); scanned using a TissueFAXS Slides system (TissueGnostics). Quantification was done using StrataQuest Analysis Software (TissueGnostics). Regressed (*n* = 5) and healthy (*n* = 5) mouse mammary gland tissue sections, 70 mammary ducts per section, were analyzed to obtain the total cell count (nuclei‐DAPI), count of GFP‐positive cells, and GFP mean intensity in the GFP‐positive cells within each duct. To obtain summary statistics for the sections, the cell counts were summed across all ducts of a section and the section mean of the duct‐mean GFP intensities was taken. Mann–Whitney test was used to calculate significance in the comparison of regressed and healthy mammary gland tissue sections.

#### Immunohistochemistry

MT‐CO1 antibody (Abcam, ab14705) staining was done on FFPE tissue sections following the standard IHC protocol, with modifications from M.O.M. kit protocol:

1. Deparaffinization—wash paraffin‐embedded tissue sections gradually in: Xylenes (Sigma‐Aldrich, 1.08298) and decreasing concentrations of Ethanol (100–96–70%) and then rinse slides in running water for 5 min. Wash in PBS‐Tween (Sigma‐Aldrich, P9416) 0.1% for 10 min.
2. Antigen retrieval—place slides in prepared citric acid‐based antigen unmasking solution (Vector Laboratories, H‐3300) in 250 ml of water and boil for 30 min in a pre‐heated steamer or 20 min in a microwave. Let the slides cool at room temperature, below 50°C, then rinse in running water for 5 min.
3. Permeabilization—incubate slides in PBS‐Triton X‐100 0.3% (Sigma‐Aldrich, T8787) 10 min and then in PBS‐Tween (0.1%) for 10 min.
4. Inactivation of endogenous hydrogen peroxidase activity—incubate slides with prepared 3% H_2_O_2_ aqueous solution from 30% stock (Sigma‐Aldrich, H1009) for 10 min, rinse in PBS 2×, 10 min.
5. Blocking: prepare and use Blocking Reagent solution from M.O.M. kit (VECTASTAIN Elite ABC‐HRP Kit, Peroxidase, Mouse IgG, Vector Laboratories, PK‐6102) and incubate for 1 h.
6. Incubate slides with M.O.M. Diluent (from M.O.M. kit) for 5 min; tip‐off diluent and incubate with primary antibody (MT‐CO1 diluted 1:500 in M.O.M. Diluent) at 4°C overnight.
7. Rinse the slides for 5 min 2×, using 1× PBS and incubate with biotinylated secondary antibody solution (Anti‐Mouse IgG reagent in M.O.M. Diluent, prepared according to kit protocol) for 10 min at room temperature.
8. Wash in PBS for 2 min, 2×. Incubate with ABC reagent (prepared by protocol from M.O.M. kit) for 30 min at room temperature. Rinse in PBS for 5 min, 2×.

Detection was done using DAB Peroxidase (HRP) Substrate Kit (Vector Laboratories, SK‐4100). Counter‐staining was done using Hematoxylin QS (Vector laboratories, H‐3404), after which the sections were dehydrated, mounted with DPX Mountant for histology (Sigma‐Aldrich, 06522), and analyzed using LMD 7000 microscope (Leica Microsystems) equipped with Leica DFC310FX digital color camera and LASV3.7 (Leica Microsystems) software.

#### RNA collection and extraction

The RNA was harvested from a pool of two 3D gels per experimental condition using 900 µl of mirVana lysis buffer. The RNA extraction was done using a mirVana miRNA Isolation Kit with phenol (Ambion, AM1560). After assessing the RNA quality and concentration on an Agilent 2100 Bioanalyzer (G2939BA), the cDNA libraries were prepared using Illumina's TrueSeq library preparation kit and a Beckman Biomek FX laboratory automation workstation. The libraries were multiplexed and sequenced in the Genomics Core Facility at EMBL Heidelberg on an Illumina HiSeq 4000 as well as an Illumina NextSeq 500 platform, generating strand‐specific single end reads with a read length of 75 or 85 bp, respectively.

#### Analysis of raw RNA sequencing data

After assessing the quality of the raw RNA sequencing reads by FastQC version 0.11.3 (Andrews, 2010), adapter trimming using Cutadapt version 1.9.1 (Martin, 2011) with default options providing the standard Illumina TrueSeq Index adapters was done. FaQCs version 1.34 (Lo & Chain, 2014) was used for subsequent quality trimming and filtering applying the following parameters: ‐q 20 ‐min_L 30 ‐n 5 ‐discard 1. The total reads per sample after trimming and filtering ranged from 34.1 to 52.0 million. Sequencing reads were aligned to the *M. musculus* reference genome (GRCm38.p4) (NCBI, 2015), which included the sequence for human cMYC and rat HER2, using Tophat2 version 2.0.10 (Hamdi *et al*, 2017) with the following parameters: ‐G ‐T ‐x 20 ‐M ‐‐microexon‐search ‐‐no‐coverage‐search ‐‐no‐novel‐juncs ‐‐mate‐std‐dev 100 ‐r 50 ‐‐min‐segment‐intron 20 ‐i 30 ‐a 6. Gene‐level count tables were obtained using the count script of the HTSeq python library version 0.6.1p1 (Anders *et al*, 2015) with default options. Only reads with unique mappings were considered. All remaining reads mapped in total to 19,500 to 20,800 genes across all samples. For performing dimensionality reduction by principal component analysis (PCA) and hierarchical clustering, normalized rlog transcript counts were utilized, which had been transformed with the “rlog” function of the Bioconductor package DESeq2 version 1.12.4 (Love *et al*, 2014). R version 3.3.1 (R‐Core‐Team, 2016) was used for conducting biostatistical analyses.

#### Differential expression analysis

The statistical analysis for differential expression was mainly done with the Bioconductor package DESeq2 version 1.12.4 (Love *et al*, 2014). Size‐factor‐based normalization was performed to control for batch effects and inter‐sample variability. Genes with less than 10 counts across all samples were filtered to increase the sensitivity of the detection of differential gene expression. Package defaults were used for dispersion estimation and differential expression analysis with the function “Deseq”, which includes independent filtering, cooks cutoff (Anders & Huber, 2010) for outlier detection and the performance of a Wald test. The animal was included as a confounder variable in the model design. Subsequently, adjusted *P*‐values were computed from the DESeq2 calculated *P*‐values by applying a Bonferroni correction for multiple testing. Genes with a *P*_adj_‐value < 0.1 were considered as significantly differentially expressed (DE). R version 3.3.1 (R‐Core‐Team, 2016) was used for conducting biostatistical analyses.

#### Gene set enrichment analysis

2,039 differentially expressed genes in residual (compared to never induced control; *q*‐value < 0.1) and 6,411 differentially expressed genes in tumor cells (compared to never induced, *q*‐value < 0.1) were taken for gene ontology (GO) enrichment analysis. GO enrichment analysis was performed using Fisher's exact test with a foreground of all respective differentially expressed genes and a background, which was composed of a unique set of five randomly picked genes per foreground gene exhibiting a similar expression mean over all samples. The analysis was done separately for upregulated and downregulated genes. The chosen cutoff for significant GO terms was *P*‐value < 0.001. Further, a gene set enrichment analysis of significantly enriched KEGG pathways was performed using the function “gage” of the likewise called R package with version 2.32.1 (Luo *et al*, 2009). The function was utilized to calculate sample‐wise test statistics with an unpaired one‐sided two‐sample *t*‐test using annotations from “org.Mm.eg.db” version 3.7.0. (Carlson, 2018). KEGG pathways with a *P*‐value < 0.05 were considered significantly enriched. Log_2_ fold changes from the differential gene expression analysis (normal/healthy in comparison with residual/regressed) were used as an input for the test.

Additionally, a reporter metabolite analysis was performed to identify metabolites or metabolic pathways that are likely to be de‐regulated. Therefore, the *q*‐values and log_2_ fold changes (FC) of the respective differentially expressed genes were used to calculate *P*‐values from a theoretical null distribution (10,000 permutations) utilizing the reporter metabolite algorithm from the piano R package (Varemo *et al*, 2013). Multiple testing adjustment was applied using the Benjamini–Hochberg procedure. The threshold for significance was *P*_adj_‐value < 0.01 for the non‐directional class, the distinct‐directional class, and the mixed‐directional class, but maximally the top 5% of the total list of tested metabolites considering each class equally. Pathway enrichment was calculated for gene sets of 1 gene per group or bigger. The gene set was obtained from a revised version of the HMR2 model (Mardinoglu *et al*, 2014), whose gene–protein–reaction annotations were translated to mouse orthologs with the Bioconductor package biomaRt version 2.38.0 (Durinck *et al*, 2009) using the ensembl database with the archived human and mouse datasets version “jul2016.archive.ensembl.org”. R version 3.3.1 (R‐Core‐Team, 2016) was used for conducting biostatistical analyses.

#### Footprinting‐based analysis of transcription factor activities

Transcription factor activities were assessed with a footprinting‐based enrichment method using the gene expression of TF targets as an estimate for TF activity. The “dorothea” collection (available on bioconductor, version 1.1.2) of mouse regulons (regulated target genes) was used with confidence levels “A” and “B” as TF‐target gene association input (Holland *et al*, 2020). The Wald statistic (two‐sided Wald test) of all genes of the respective comparison between populations was used as a gene statistic input for the msVIPER algorithm to calculate the (enrichment‐based) TF activity score with parameters set to minsize = 4 and ges.filter = F. Alternatively, the genome‐wide normalized rlog‐transformed transcript counts (described in “Analysis of raw RNA sequencing data”) were used as input for the VIPER algorithm to calculate a TF enrichment of the residual cell population against a combined normal and tumor population using the following parameters: minsize = 4, ges.filter = F, method = “ttest”. Subsequently, a two‐sided Student's *t*‐test was performed to calculate each TF's Student *t*‐statistic and *P*‐value as a proxy for activity. The R package viper version 1.24.0 was used for the “MsViper” and “viper” algorithms (Alvarez *et al*, 2016). For both analyses, TFs with a Benjamini–Hochberg‐adjusted *P* ≤ 0.01 were considered significant. The Bioconductor package biomaRt version 2.38.0 (Durinck *et al*, 2009) using the archived ensembl mouse dataset version "jul2016.archive.ensembl.org" was used to translate ensemble gene ids to mgi gene symbols. R version 3.3.1 (R‐Core‐Team, 2016) was used for conducting biostatistical analyses.

#### Integration of transcriptomic data into flux balance analysis

Differential gene expression data were integrated into flux balance analysis (FBA) using a new simulation method, metabolic analysis with relative gene expression (MARGE). This method aims to overcome some limitations identified in other previously published methods (Machado & Herrgard, 2014). In particular, it avoids making assumptions on any direct proportionality between transcript levels and reaction rates; instead, it uses relative expression between two conditions, as an indication of the direction and magnitude of the flux control exerted on a metabolic pathway through transcriptional regulation. The implementation is based on a previously proposed extension of FBA that integrates gene–protein–reaction (GPR) association rules into the stoichiometric matrix of the metabolic network, allowing the computation of enzyme‐specific flux rates (Machado *et al*, 2016), and is formulated as two‐step linear optimization problem. The first step optimizes the agreement between relative enzyme usage and relative gene expression, and the second adds a parsimonious enzyme usage criterion. Metabolite measurements can optionally be input as additional relative (between experimental conditions) or absolute constraints (for each experimental condition separately) at this step of optimization.

Step 1:

$$\begin{array}{c} \min\;{\text{obj}}_{1}=\sum_{i=1}^{n}\left|u_{i}^{b}‐\frac{e_{i}^{b}}{e_{i}^{a}}u_{i}^{a}\right|\\ s\text{.t}.\\ \;S^{\text{ext}}\cdot \left|\begin{array}{c} v^{a}\\ u^{a} \end{array}\right|=0\\ \;S^{\text{ext}}\cdot \left|\begin{array}{c} v^{b}\\ u^{b} \end{array}\right|=0\\ \;lb^{a}<v^{a}<ub^{a}\\ \;lb^{b}<v^{b}<ub^{b}\\ \;u_{i}^{a}>u_{\min}\;\text{if}\;e_{i}^{a}>0\forall i\\ \;u_{i}^{b}>u_{\min}\;\text{if}\;e_{i}^{b}>0\forall i \end{array}$$

Step 2:

$$\begin{array}{c} \min\;\sum_{i=1}^{n}\left|u_{i}^{a}\right|+\sum_{i=1}^{n}\left|u_{i}^{b}\right|\\ s\text{.t}.\\ \;(\text{all}\;\text{of}\;\text{the}\;\text{above})\\ \;\sum_{i=1}^{n}\left|u_{i}^{b}‐\frac{e_{i}^{b}}{e_{i}^{a}}u_{i}^{a}\right|<{\text{obj}}_{1}\left(1+\varepsilon \right) \end{array}$$

where *a* and *b* are two experimental conditions, *e^a^* and *e^b^* are the gene expression in each condition, *v^a^* and *v^b^* are reaction flux vectors, *u^a^* and *u^b^* are enzyme usage vectors, *S* ^ext^ is the extended stoichiometric matrix, *u*_min_ is a flux activation threshold (set to 0.001), and *ɛ* is a relaxation term with regard to the first objective (set to 0.1).

#### Genome‐scale metabolic modeling

Transcriptomic and extracellular GC‐MS metabolomic data from this study were used as model inputs to refine the phenotype predictions performed with flux balance analysis. The transcriptomic data were provided as log_2_ fold changes in significantly differentially expressed metabolic genes (*q*‐value < 0.1) between the two experimental conditions. The metabolomic data were used to constrain the metabolite uptake/secretion rates in the model, both in terms of absolute rates per condition, and relative rates between conditions. The fold change in significantly changed extracellular metabolite profiles (*P*_adj_‐value < 0.001) between conditions was calculated and imposed as relative constraints in the model with a deviation tolerance of 50% to account for measurement errors. The media base line was therefore subtracted from the measurements of the experimental conditions. The measurements of the pure medium additionally allowed the distinction between an active secretion into the medium or an uptake from the medium for all conditions. Thus, for all conditions, for which the metabolite levels changed significantly in comparison to the media, absolute uptake/secretion constraints (1% of the maximum uptake rate) were imposed to ensure a minimum level of metabolite uptake/secretion in accordance with the data.

A revised version of the human genome‐scale metabolic model HMR2 (Mardinoglu *et al*, 2014) was used for simulations. The gene–protein–reaction (GPR) annotations were translated to mouse gene orthologs with the Bioconductor package biomaRt version 2.38.0 (Durinck *et al*, 2009) using the ensembl database with the archived human and mouse datasets version "jul2016.archive.ensembl.org". The GPR annotations as well as the model itself had been updated and corrected to yield more accurate flux predictions. In brief, this included the introduction of a mitochondrial intra‐membrane space (adapted from Swainston *et al*, 2016) to improve the prediction of respiratory ATP synthesis, the revision of reactions from the beta‐oxidation pathway and auxiliary enzymes, the introduction of ATP maintenance costs, and the adaption of model uptakes and releases of metabolites from experimental data (Jain *et al*, 2012). Further, atomically unbalanced reactions were removed and the directionality of reactions was constraint where infeasible.

The import/export of SBML files was obtained through the libSBML API (Bornstein *et al*, 2008) using the load_cbmodel of reframed. The simulations were performed using the *ReFramed* python package version 1.0.0 (https://github.com/cdanielmachado/reframed). In particular, we used the MARGE function (implementation of the method described above) with the following parameter settings: growth_frac_a=0.8, growth_frac_b=0.8, activation_frac=0.001, step2_tol=0.1. The IBM ILOG CPLEX Optimizer version 12.8.0 was used for solving the MILP problems. All simulations were conducted with Python 3.6.9.

The metabolic model, the modeling parameters, and the analysis can be found on (https://github.com/katharinazirngibl/MinimalResidualDisease/).

#### Human breast cancer transcriptome comparison

Microarray gene expression datasets from breast cancer patients pre‐ and post‐treatment (Gonzalez‐Angulo *et al*, 2012) and control breast tissue from healthy women (Maire *et al*, 2013) were downloaded from Gene Expression Omnibus (GEO) (Cong *et al*, 2013). Each dataset was first analyzed independently, which included filtering for sample outliers, normalization, background correction, minimal intensity filtering of genes, and the annotation of genes from probe set IDs with the removal of multiple mappings of transcript cluster identifiers. The sample outliers were identified with the function “arrayQualityMetrics” of the likewise called R package with version 3.38.0 (Kauffmann *et al*, 2009). Normalization and background correction were done using the “rma” function of the R package oligo version 1.46.0 carvalho (Carvalho & Irizarry, 2010). The minimal intensity threshold for gene filtering was determined by fitting a null model to the whole data set and taking the lower 5% boarder as a cutoff. The two datasets were then combined and processed together a second time (normalization, outlier removal, intensity filtering). In order to address the batch effect of the joined data stemming from the two experimental settings, the first principal component was removed from the data set. In addition, the “normal‐like” tumor subtype of the patients' dataset was removed due to the poorly defined diagnostic category and high biological variability. For the differential gene expression, a gene‐wise linear model was fitted to the dataset using generalized least squares and including the tumor subtype as a confounder variable if applicable. Next, an empirical Bayes moderated *t*‐statistic and log‐odds were computed with the “eBayes” function of the limma package version 3.38.3 (Smyth, 2004; Ritchie *et al*, 2015) using the package defaults. All genes with a Benjamini–Hochberg‐adjusted *P*‐value < 0.1 were considered differentially expressed. For the joint clustering with the mouse transcriptome data, the mouse genes were translated to human orthologs using the Bioconductor package biomaRt version 2.38.0 (Durinck *et al*, 2009) and the ensembl database with the archived mouse and human datasets version "jul2016.archive.ensembl.org". R version 3.3.1 (R‐Core‐Team, 2016) was used for conducting biostatistical analyses.

#### Intracellular and extracellular sample harvest and metabolite extraction from 3D organoids

For metabolomic experiments, matrix (volume of 100 µl) was mixed with 12,000 primary mouse mammary epithelial cells and dispensed into flat bottom wells (CellBIND 12 Well Clear Multiple Well Plates, Corning 3336). Cells were cultivated for 7 days, with media change on every second day, before oncogene induction. Upon that point, media was changed daily for all the three conditions (normal, tumor, and residual). Timepoints of induction and de‐induction were designed to allow collection and processing of the samples from normal, tumor, and residual organoids simultaneously. The samples (two technical replicates, per condition) were pooled, initially from 6, then from 3 wells, as the signal was strong enough to quantify metabolites. Prior to the harvest of organoids for metabolomics, 50 µl of spent growth media was snap‐frozen and stored at −80°C until the extraction of metabolites for extracellular metabolomics. Subsequently, the organoid structures were freed from the Matrigel by adding liberase and collagenase for 1.5 h at 37°C to the remaining medium. Following this incubation, the medium was aspirated, and the organoids were washed with room temperature PBS and centrifuged (1,000 r.p.m., 2 min, room temperature). This step was performed three times until the addition of 200 µl cold (−80°C) HPLC‐grade methanol (Biosolve Chimie, 136841). For metabolite extraction, adonitol (Alfa Aesar, 488‐81‐3) was added as an internal standard to the mixture of organoids/methanol. The samples were incubated at 72°C for 15 min followed by addition of 200 μl ice‐old Milli‐Q water. Following centrifugation (12,500 *g*, 10 min, 4°C), the supernatants were collected and dried using a GeneVac EZ‐2 plus evaporating system (SP Scientific). The dried metabolite extracts were stored at −80°C until metabolomic analysis. Metabolite extraction from the spent growth media was performed as described above by adjusting the volume of the extraction solvents to 100 µl of HPLC‐grade methanol and 100 µl of Milli‐Q water. Finally, 50 µl from the initial pure growth medium and from the last washing step with PBS were collected and extracted as the spent growth media samples. The last washing step was employed as control to validate the effective washing of the organoids from the extracellular medium before quenching.

For experiments following extracellular glucose change after 3 BP treatment, seeding, cell cultivation, oncogene induction, media change, and harvesting for metabolic analyses were done as described above. Prior to collection, cells were incubated for 5 h with 3‐BP, at the doses 50 and 250 µM. Samples from four and two biological replicates were analyzed for unlabeled and labeled experiments, respectively.

#### In vivo and ex vivo mammary glands experiments

For experiments that allowed for tumorigenesis and regression *in vivo*, food pellets supplemented with doxycycline (625 mg/kg) were used for tumor induction in mice, which were weekly monitored for tumor detection and their overall health. Full blown tumors developed in the period of 4–6 weeks and when burden was too large (*d* = 2 cm), animals were given food without doxycycline which resulted in the fast tumor regression to a non‐palpable state. At the timepoint of the complete tumor regression (9 weeks after oncogenes deactivation), mammary glands were harvested from these mice, along with the wild‐type (non‐inducible) siblings, which had the same treatment. Before harvesting, vaginal lavage to check for the phase of estrous cycle was obtained, according to the modified protocol (McLean *et al*, 2012): Slides were dried at room temperature, fixed in 10% formalin, washed in 1× PBS, stained with crystal violet solution (Sigma, V5265) followed by wash in water and analyzed using Leica Application Suite X and Leica DFC7000 T microscope (Leica Microsystems).

For the [U‐^13^C]glucose tracing experiment, the harvested mammary glands were dissected, minced, and digested for 1.5–2 h at 37°C using collagenase and liberase enzymes, then cultured for 8 h at 37°C in 5% (vol/vol) CO_2_ atmosphere, in DMEM glucose‐ and pyruvate‐free medium (Thermo Fisher Scientific, 11966025) supplemented with 4.5 g/l labeled d‐glucose U‐^13^C, 99% (Cambridge Isotope Laboratories, Inc., CLM‐1396) and components from MEGM BulletKit (Lonza, CC‐3150). For the non‐labeled metabolomic experiment, the harvested mammary glands were dissected and cultured for 8 h at 37°C in 5% (vol/vol) CO_2_ atmosphere, in DMEM, high glucose (4.5 g/l glucose) GlutaMAX (Gibco, 10569044) supplemented with components from MEGM BulletKit (Lonza, CC‐3150). For both the isotope tracing and unlabeled experiments, 50 µl from the spent growth media was collected following the 8 h of incubation period. For intracellular metabolomics, the mammary glands were collected following the 8 h of incubation period, washed with room temperature PBS, and centrifuged (1,000 r.p.m., 2 min, room temperature). The washing procedure was performed three times before the addition of 200 µl cold (−80°C) HPLC‐grade methanol. Subsequently, the metabolite extraction was performed as described in “Intracellular and extracellular sample harvest and metabolite extraction from 3D organoids”.

#### Targeted metabolomic analysis with gas chromatography—mass spectrometry

Dried metabolite extracts were derivatized with 50 µl of 20 mg/ml methoxyamine hydrochloride (Alfa Aesar, A19188) solution in pyridine (Sigma‐Aldrich, 437611) for 90 min at 40°C, followed by addition of 100 µl *N*‐methyl‐trimethylsilyl‐trifluoroacetamide (MSTFA; Alfa Aesar, A13141) for 12 h at room temperature (Kanani & Klapa, 2007; Kanani *et al*, 2008). GC‐MS analysis was performed using a Shimadzu TQ8040 GC‐(triple quadrupole) MS system (Shimadzu Corp.) equipped with a 30 m × 0.25 mm × 0.25 μm ZB‐50 capillary column (7HG‐G004‐11; Phenomenex). One microliter of sample was injected in split mode (split ratio = 1:10) at 250°C using helium as a carrier gas with a flow rate of 1 ml/min. GC oven temperature was held at 100°C for 4 min followed by an increase to 320°C with a rate of 10°C/min, and a final constant temperature period at 320°C for 11 min. The interface and the ion source were held at 280 and 230°C, respectively. The detector was operated both in scanning mode (recording in the range of 50–600 *m*/*z*) and in MRM mode (for specified metabolites). For peak annotation, the GC‐MS solution software (Shimadzu Corp.) was utilized. The metabolite identification was based on an in‐house database with analytical standards being utilized to define the retention time, the mass spectrum, and marker ion fragments for all the quantified metabolites. The metabolite quantification was carried out by integrating the area under the curve of the MRM transition of each metabolite. The data were further normalized to the area under the curve of the MRM transition of adonitol and to total metabolite levels. To identify the statistically significant altered metabolites, the limma package (Ritchie *et al*, 2015) (version 3.36.5) in R (version 3.5.2) was utilized with the significance threshold corresponding to a Benjamini–Hochberg adjusted *P*‐value ≤ 0.05.

#### Isotope tracing analysis

For the [U‐^13^C] glucose tracing experiments, dried metabolite extracts were derivatized with 50 µl of 20 mg/ml methoxyamine hydrochloride (Alfa Aesar, A19188) solution in pyridine (Sigma‐Aldrich, 437611) for 90 min at 40°C, followed by addition of 100 µl *N*‐tert‐Butyldimethylsilyl‐*N*‐methyltrifluoroacetamide + 1% tert‐Butyldimethylchlorosilane (Sigma‐Aldrich, 00942) for 1 h at 60°C. The samples remained at room temperature for 5 h and then analyzed by GC‐MS. The GC‐MS was operated as described in “Targeted metabolomic analysis with gas chromatography—mass spectrometry” with the following difference: GC oven temperature was held at 100°C for 3 min followed by an increase to 300°C with a rate of 3.5°C/min and a final constant temperature period at 300°C for 10 min. The detector was operated in single ion monitoring (SIM) mode for the ion fragments *m*/*z* 261, 262, 263, and 264 which correspond to all possible mass isotopologues of lactate. Mass isotopologue distributions were determined by integrating the area under the curve of the ion fragments. The data were corrected for natural isotope abundance using the Isotope Correction Toolbox (ICT) (Jungreuthmayer *et al*, 2016). Significance was calculated by unpaired two‐sample *t*‐test following assessment of normality and equal variance using the Shapiro–Wilk's test and F test, respectively.

#### Untargeted metabolomics by flow injection mass spectrometry

Untargeted metabolomic analysis was performed based on a previously published approach (Fuhrer *et al*, 2011). Briefly, samples were analyzed on a LC‐MS platform consisting of a Thermo Scientific Ultimate 3000 liquid chromatography system with autosampler temperature set to 10°C coupled to a Thermo Scientific Q‐Exactive Plus mass spectrometer equipped with a heated electrospray ion source and operated in negative ionization mode. The isocratic flow rate was 150 μl/min of mobile phase consisting of 60:40% (v/v) isopropanol:water buffered with 1 mM ammonium fluoride at pH 9 and containing 10 nM taurocholic acid and 20 nM homotaurine as lock masses. Mass spectra were recorded in profile mode from 50 to 1,000 *m*/*z* with the following instrument settings: sheath gas, 35 a.u.; aux gas, 10 a.u.; aux gas heater, 200°C; sweep gas, 1 a.u.; spray voltage, −3 kV; capillary temperature, 250°C; S‐lens RF level, 50 a.u; resolution, 70k @ 200 *m*/*z*; AGC target, 3 × 10^6^ ions, max. inject time, 120 ms; and acquisition duration, 60 s. Spectral data processing was performed using an automated pipeline in R. Detected ions were tentatively annotated as metabolites based on matching accurate masses of assumed [M‐H] and [M‐2H] ions with either no, one, or two ^12^C to ^13^C exchanges within a tolerance of 5 mDa to compounds in the Human Metabolome database as reference (Wishart *et al*, 2018), with the method‐inherent limitation of being unable to distinguish between isomers. Hierarchical clustering of the ions was performed with the complete linkage method and the Euclidean distance as a distance metric. Only ions with non‐zero intensity and unique annotation including no assumed mass shift were used for the clustering analysis. For the visualization, ions with a false discovery rate < 0.05 as determine by unpaired two‐sided *t*‐tests and subsequent multiple hypothesis testing correction according to Storey's and Tibshirani's method (Storey & Tibshirani, 2003) were considered as significantly changed.

#### Lipidomics

Acidic extractions were performed as described (Ozbalci *et al*, 2013) in the presence of an internal lipid standard mix containing 50 pmol phosphatidylcholine (13:0/13:0, 14:0/14:0, 20:0/20:0; 21:0/21:0, Avanti Polar Lipids), 50 pmol sphingomyelin (d18:1 with *N*‐acylated 13:0, 17:0, 25:0), 100 pmol D6‐cholesterol (Cambridge Isotope Laboratory), 25 pmol phosphatidylinositol (16:0/ 16:0, Avanti Polar Lipids), 25 pmol phosphatidylethanolamine and 25 pmol phosphatidylserine (both 14:1/14:1, 20:1/20:1, 22:1/22:1), 25 pmol diacylglycerol (17:0/17:0, Larodan), 25 pmol cholesteryl ester (9:0, 19:0, Sigma), 24 pmol triacylglycerol (D5‐Mix, LM‐6000/D5‐17:0/17:1/17:1, Avanti Polar Lipids), 5 pmol ceramide and 5 pmol glucosylceramide (both d18:1 with *N*‐acylated 15:0, 17:0, 25:0), 5 pmol lactosylceramide (d18:1 with *N*‐acylated C17 fatty acid, Avanti Polar Lipids), 10 pmol phosphatidic acid (21:0/22:6, Avanti Polar Lipids), 10 pmol phosphatidylglycerol (14:1/14:1, 20:1/20:1, 22:1/22:1), 10 pmol lyso‐phosphatidylcholine (17:1, Avanti Polar Lipids), 50 pmol cardiolipin (14:1/14:1/14:1/15:1, Avanti Polar Lipids), and 50 pmol monolysocardiolipin (16:0/16:0/16:0, Avanti Polar Lipids). Neutral extractions were performed as described (Ozbalci *et al*, 2013) containing a phosphatidylethanolamine plasmalogen (PE P‐)‐standard mix which was spiked with 16.5 pmol PE P‐Mix 1 (16:0p/15:0, 16:0p/19:0, 16:0p/ 25:0), 23.25 pmol PE P‐Mix 2 (18:0p/15:0, 18:0p/19:0, 18:0p/25:0), and 32.25 pmol PE P‐Mix 3 (18:1p/15:0, 18:1p/19:0, 18:1p/25:0). Lipid standard preparations were done as described in Ozbalci *et al* (2013). Lipid extracts were resuspended in 60 µl methanol, and samples were analyzed on a QTRAP 6500+ mass spectrometer (Sciex) with chip‐based (HD‐D ESI Chip, Advion Biosciences, USA) nano‐electrospray infusion and ionization via a Triversa Nanomate (Advion Biosciences, Ithaca, USA) as previously described (Ozbalci *et al*, 2013; Mucksch *et al*, 2019). Data evaluation was done using LipidView (Sciex) and an in‐house‐developed software (ShinyLipids). Since triacylglycerol (TAG) could not be reliably measured, it was excluded from the downstream analysis.

#### NOS enzymatic assay

Mammary glands were dissected and homogenized in NOS assay buffer and further processed following the Nitric Oxide Synthase Activity Assay Kit (Abcam, ab211083) protocol for measuring enzymatic activity of nitric oxide synthase (NOS). Statistics were calculated by unpaired two‐sample *t*‐test followed by the assessment of normality and equal variance using the Shapiro–Wilk's test and *F* test, respectively.

#### Glycolysis inhibition experiments

Cells were seeded in 3D conditions (5 µl of 80% Matrigel droplet) in black TC‐treated imaging 96‐well plates (Falcon, 353219) with clear flat bottom. 3‐bromopyruvate (Sigma‐Aldrich, 16490) was added to the cell medium, whereby the following doses (µM) were tested: 250, 50, 25, 10, 2, 0 (vehicle, water). A CellTox Green Cytotoxicity Assay (Promega, G8741) was performed according to the manufacturer instructions to measure cell death after 72 or 48 h of treatment. Green fluorescence was measured using an EnVision plate reader (PerkinElmer), Resorufin/Amplex Red FP 535 for excitation, and an Europium 615 emission filter. Data analysis was performed using GraphPad Prism 8, and fold changes were calculated by normalization to the untreated control using a transform function. *Y* values were transformed using *Y* = *Y*/*K* with a different *K* for each dataset and *K* being the mean of the untreated control for normal, tumor, and residual separately. For quantification of cell death of normal, tumor, and residual cells after 72‐h treatment with 3‐BP, two‐way ANOVA test was used to calculate the significance. Tukey HSD was computed for multiple pairwise comparisons between the means of the groups. For cell death quantification of passaged residual and normal cells after 48 h of treatment with 3‐BP, significance was calculated using multiple *t*‐tests, one unpaired *t*‐test per row.

All experiments were reproduced two or three times; the number of biological replicates is depicted in the figure legends, and for each biological replicate, there were 5–6 technical replicates of which an average was taken.

Images were taken over the time‐course of the experiment using the high‐throughput Olympus ScanR microscope in transmission mode. Each well of the 96‐well plate was imaged using 1 ROI with 21 Z‐stacks (100 µm distance between the stacks) at 4× magnification in a chamber with standard conditions (37°C, 5% CO_2_). The projections of z‐stacks and image stitching were done using the Fiji software.

#### DNA methylation profiles

The cells for the DNA extraction were harvested from three 3D gels per experimental condition by the digestion of the matrix with collagenase and liberase for 1.5 h at 37°C. Subsequently, the individually digested gels were pooled, washed three times with PBS and STOP medium (Dulbecco's modified eagle medium:F12, DMEM:F12 with HEPES (15 mM), 1:1 mixture with 3.151 g/l glucose, with l‐glutamine, supplemented with HEPES to the final concentration of 25 mM and 10% of fetal bovine serum, tetracycline free) and centrifuged shortly (1,000 r.p.m., 2 min, at room temperature). The DNA extraction from the harvested cells was done following the Qiagen protocol for cultured cells (Qiagen Blood & Cell Culture DNA Mini kit, 13323). The libraries for enzymatic methyl‐seq (EM‐seq) were prepared with the NEBNext enzymatic Methyl‐seq Kit (NEW ENGLAND BioLabs Inc.) from 100 ng of sheared DNA. According to the protocol's recommendations, 2 ng of lambda phage DNA and 0.1 ng of pUC19 plasmid DNA were spiked‐in per 100 ng sample DNA prior to fragmentation. The fragmentation of the DNA mix was done using a Covaris S2‐focused ultrasonicator. Bisulfite‐free NEBNext EM‐seq libraries were sequenced in the Genomics Core Facility at EMBL Heidelberg on an Illumina HiSeq 2500 platform generating directional paired‐end reads with a read length of 135 bp. Each sample was sequenced on a single lane. The total reads per sample ranged from 229.8 to 250.6 million. The sequencing reads were aligned with the “qAlign” function of the R package QuasR version 1.22.1 (Gaidatzis *et al*, 2015) using the reference genome sequence for *M. musculus* from the R package BSgenome.Mmusculus.UCSC.mm10 version 1.4.0, (The‐Bioconductor‐Dev‐Team, 2014) the R bowtie wrapper Rbowtie version 1.22.0 (Hahne *et al*; Langmead *et al*, 2009), and the parameter setting modifications: bisulfite=“undir”. The average coverage ranged from 9.4 to 14.5 reads per CpG site. The DNA methylation percentage was quantified genome‐wide for all cytosine nucleotides in CpG context with the function “qMeth” from QuasR using the information from both strands combined.

The visualization of methylation percentages and promoters at their genomic location was done with the R packages trackViewer version 1.26.2 (Ou & Zhu, 2019) and rtracklayer version 1.50.0 (Lawrence *et al*, 2009) as well as the annotation from the R package "TxDb. Mmusculus.UCSC.mm10.knownGene" version 3.10.0 (BC Team, 2019).

R version 3.5.1 (R‐Core‐Team, 2016) was used for conducting biostatistical analyses.

## Author contributions

KRS and AA carried out mouse work, optimized the 3D culture system, and characterized the system through immunofluorescence staining and RNA sequencing. KRS and EK optimized and performed all metabolic experiments. KZ devised and performed statistical analyses and integration of multi‐omics datasets, as well as comparisons of the mouse and human transcriptomes. DM and KZ performed genome‐scale metabolic modeling and flux balance analysis. DCS performed untargeted metabolomics analysis. KZ performed clustering and pathways analysis. EK optimized and analyzed targeted metabolomics and ^13^C tracer experiments. CL and BB performed lipidomic analyses. KRS and EK carried out the NOS enzymatic assay. KRS performed immunohistochemical and immunofluorescence staining on mouse tissue sections. KRS and SG designed and performed glycolysis‐inhibition experiments. SG analyzed CellTox and ScanR data and contributed to manuscript preparation. KRS collected and extracted DNA for methylation experiments. KZ analyzed DNA methylation data. KRS, EK, and KZ carried out study design, interpretation, and manuscript preparation. MJ and KRP provided study design, supervision, interpretation, manuscript preparation, and critical review.

## Conflict of interest

The authors declare that they have no conflict of interest.

## Supporting information



Appendix

Table EV1

## Data Availability

RNA‐seq data generated and analyzed during the current study are available at ArrayExpress (https://www.ebi.ac.uk/arrayexpress/) under accession number E‐MTAB‐8834 (http://www.ebi.ac.uk/arrayexpress/experiments/E‐MTAB‐8834). Enzymatic Methyl‐seq (EM‐seq) data generated and analyzed during the current study were submitted to ArrayExpress (https://www.ebi.ac.uk/arrayexpress/) under accession number E‐MTAB‐10979 (http://www.ebi.ac.uk/arrayexpress/experiments/E‐MTAB‐10979). GC‐MS data generated and analyzed during the current study are available at MetaboLights Database as MTBLS1507. Access: https://www.ebi.ac.uk/metabolights/MTBLS1507. Lipidomics, targeted metabolomic data, and untargeted metabolomics (FIA‐MS) data generated and analyzed during the current study are available at Mendeley (https://data.mendeley.com/datasets/8gby9dxh83/draft?a=fb9d4bd8‐0acd‐47d9‐bb27‐607b56e89d63). The human genome‐scale metabolic model HMR2 revised during the current study and used for simulation https://github.com/katharinazirngibl/MinimalResidualDisease/. Source Data for all main and Appendix figures are available at BioStudies https://www.ebi.ac.uk/biostudies/studies/S‐BSST713, Identifier: S‐BSST713.
