# Supplementary material for: Metabolic memory underlying minimal residual disease in breast cancer
Source: Mol Syst Biol. 2021 Oct 25;17(10):e10141. doi: 10.15252/msb.202010141 (PMC8543468; doi:10.15252/msb.202010141)
Supplement: Supplementary file 1 — Appendix [file MSB-17-e10141-s003.pdf]

Table of Contents

Appendix Figure S1: Effect of doxycycline concentration on the phenotype, tumor induction and mitochondrial function.
Appendix Figure S2: Transcriptome data analysis of significantly altered gene subsets shows the distinctiveness of residual cells, identifying enrichment of pathways involved in repolarization process and metabolic alterations seen in the tumor cells.
Appendix Figure S3: Set-up of metabolic methods for 3D cultures aimed at harvesting metabolites of organoid populations with an improved signal of intracellular metabolites following matrix digestion.
Appendix Figure S4: Principal component analyses (PCA) of additional omics layers reveals the resemblance of residual cells to the tumor cells.
Appendix Figure S5: Analysis of measured intra- and extracellular metabolites demonstrates similarities between residual and tumor cells regarding their central carbon metabolism.
Appendix Figure S6: Global overview of altered metabolic pathways in tumor cells compared to normal in an extended representation.
Appendix Figure S7: Global overview of altered metabolic pathways in residual cells compared to normal in an extended representation.
Appendix Figure S8: Transcript expression of genes from the Oxidative Phosphorylation (OXPHOS) pathway in tumor and residual cells.
Appendix Figure S9: Immunofluorescence staining on mouse mammary tissue sections shows higher expression of Arginase1 in residual cells of regressed tissue as compared to normal cells of healthy tissue.
Appendix Figure S10: Extended validation of transcriptional changes in patient datasets.
Appendix Figure S11: Treatment with 3-bromopyruvate functionally validates an altered glycolysis in the residual cells.
Appendix Figure S12: Transcription factor activity analysis reveals a distinct HIF1α signaling activity in residual cells.
Appendix Figure S13: DNA methylation analysis of promoter regions of HIF1, HIF1 glycolytic co-activator genes and selected HIF1α target genes shows a consistent and isoform specific demethylation in tumor and residual cell populations.
Appendix Table S1: GO terms of significantly downregulated genes.
Appendix Table S2: GO terms of significantly upregulated genes.
Appendix Table S3: Footprinting based transcription factor enrichment of the residual cell population in comparison to normal and tumor.

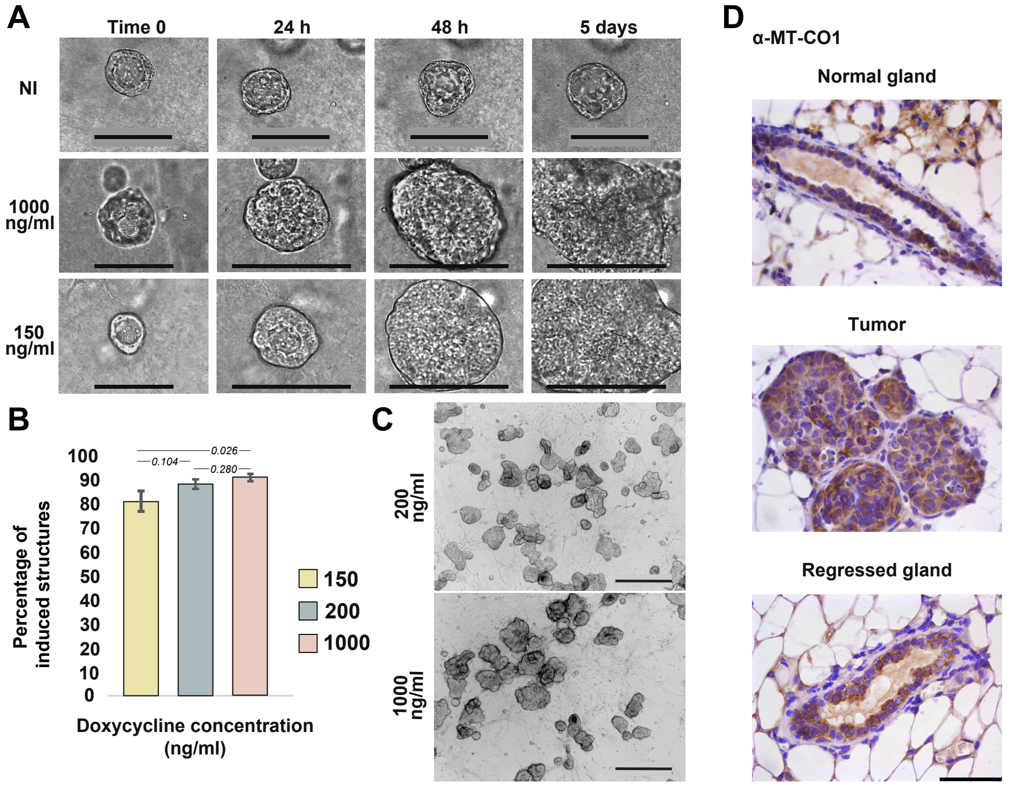

Appendix Figure S1: Effect of doxycycline concentration on the phenotype, tumor induction and mitochondrial function.

A, Representative bright field images of 3D cultures at indicated time points and doxycycline concentrations. Scale bar: 100 µm.

B, Percentage of induced structures *in vitro* (5th day on doxycycline) at the concentrations of 150-(n=2), 200-(n=3) and 1000 ng/mL (n=3). Data shown as mean ± SEM. Numbers marking comparisons show *p*-values (two-tailed t test).

C, Representative bright field images of induced structures on the 5th day on doxycycline at the concentration of 200 ng/mL (top) and 1000 ng/mL (bottom). Scale bar: 500 µm.

D, IHC on MT-CO1 protein in mouse mammary gland tissue sections. Representative images from top to bottom: normal gland from age-matched control animal, tumor from an animal at 3 weeks on doxycycline, regressed gland from an animal at 2 weeks off doxycycline. Scale bar: 50 µm.

Data information: **B**, Number of replicates corresponds to different animals.

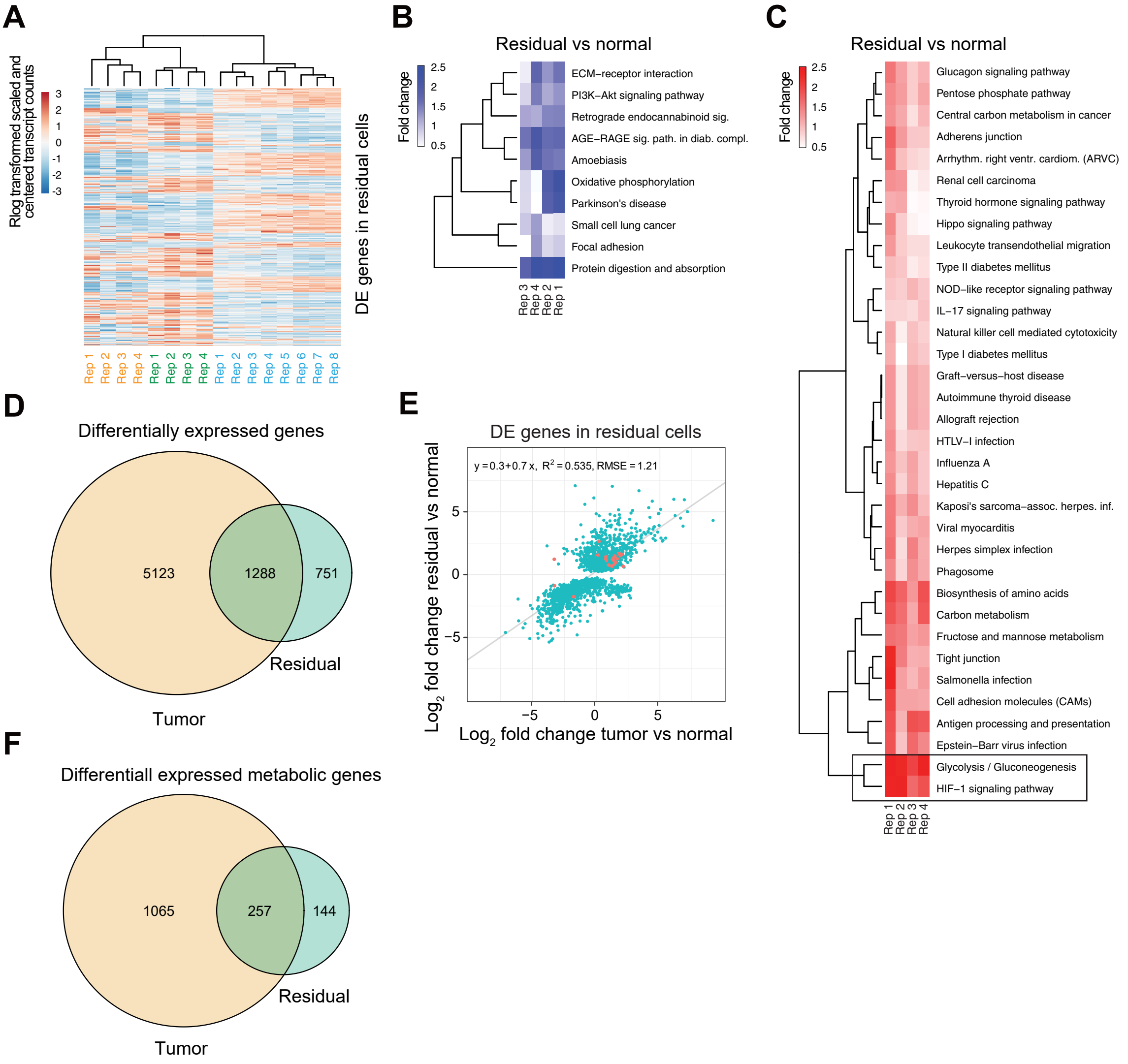

Appendix Figure S2: Transcriptome data analysis of significantly altered gene subsets shows the distinctiveness of residual cells, identifying enrichment of pathways involved in repolarization process and metabolic alterations seen in the tumor cells.

A, Heatmap with rlog transformed and gene-wise centered and scaled (stdv = 1) transcript counts of differentially expressed genes (two-sided Wald test (Love, Huber et al., 2014), Bonferroni adjusted *p*-value < 0.1) in residual cells compared to normal.

B-C, Heatmap of significantly enriched **(B)**, downregulated and **(C)**, upregulated KEGG pathways (unpaired one-side two-sample t-test, *p*-value < 0.05) of residual cells compared to normal. The enrichment was calculated from transcriptome derived $\log_2$ fold changes. Clustering is based on the sample-wise calculated test statistics of the individual KEGG pathways (Methods).

D, Overlap of differentially expressed genes in tumor and residual cell populations compared to normal.

E, Scatter plot of differentially expressed genes of the residual cell population. Log_2fold changes in comparison to normal are depicted, plotting the tumor against the residual population. Genes associated with glycolysis and the pentose phosphate pathway are highlighted in red.

F, Overlap of differentially expressed metabolic genes in tumor and residual cell populations compared to normal.

Data information: **A**-**F**, Number of replicates (normal n=8, tumor and residual n=4) corresponds to different animals. **A**-**C**, Hierarchical clustering with the complete linkage method and the Euclidean distance as a distance metric was used for clustering.

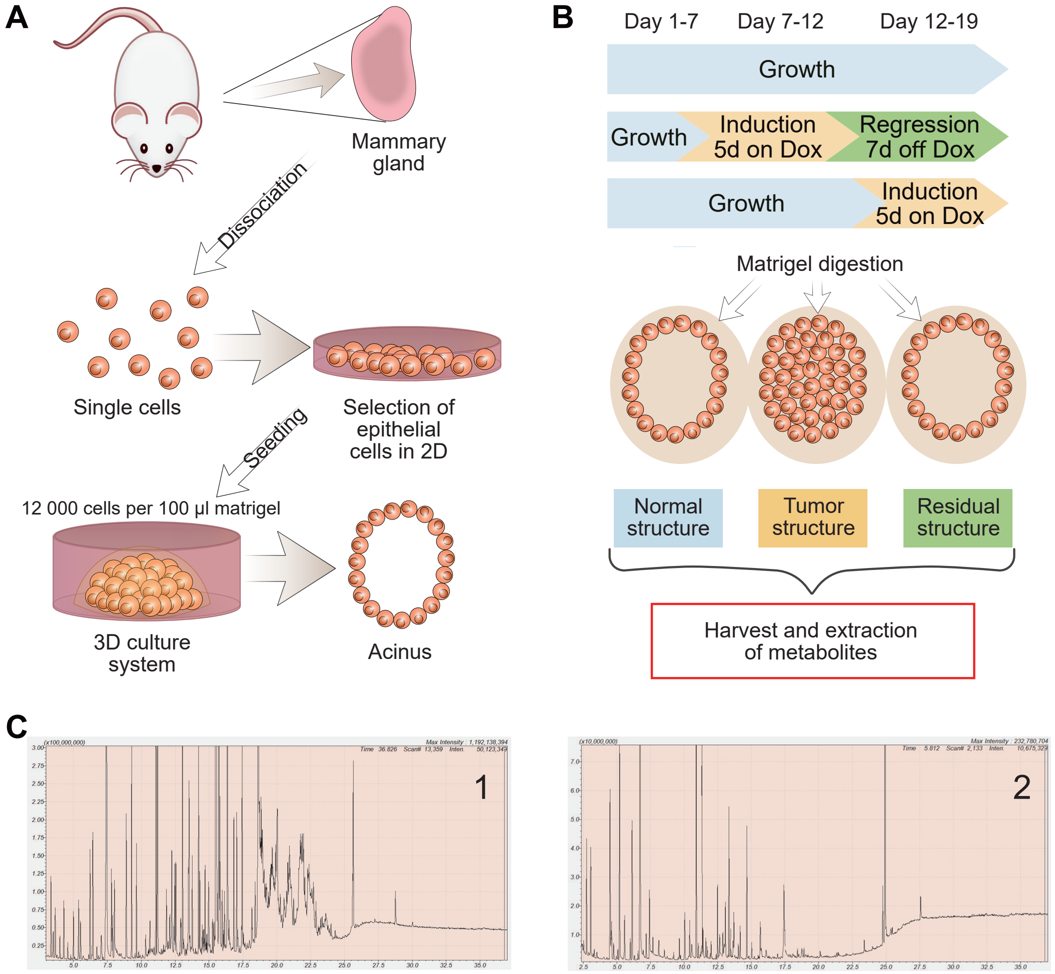

Appendix Figure S3: Set-up of metabolic methods for 3D cultures aimed at harvesting metabolites of organoid populations with an improved signal of intracellular metabolites following matrix digestion.

A, Primary epithelial cells obtained from mouse mammary glands are grown in Matrigel matrix, which enables growth in 3D and the formation of hollow acini.

B, Schematic timeline of the experiments showing the period of growth, tumor induction (5 days on doxycycline) and tumor regression (after doxycycline withdrawal, 7 days off). Experiments were designed in such a way that the metabolites from all three conditions (normal, tumor and residual) were harvested at the same endpoint after matrix gel digestion.

C, Representative total ion chromatograms showing a strong Matrigel matrix signal when no digestion is performed for the collection of structures (1), and an improved signal of intracellular metabolites after gel digestion (2).

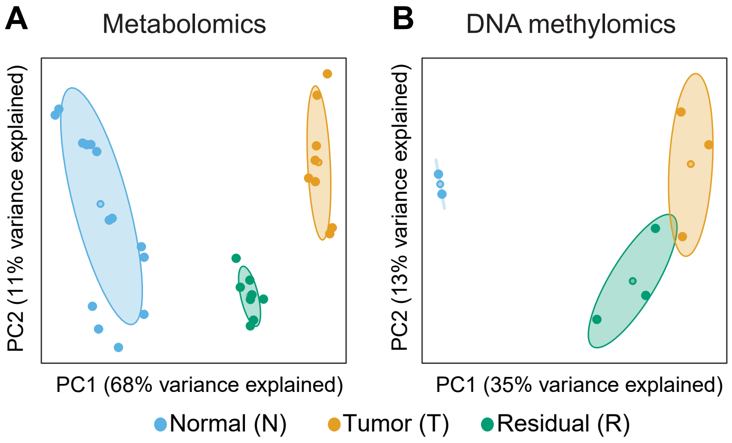

Appendix Figure S4: Principal component analyses (PCA) of additional omics layers reveals the resemblance of residual cells to the tumor cells.

A, PCA analysis of normal (blue; n=4, WT n=2), tumor (orange; n=3) and residual (green; n=3) populations based on the extracellular spent growth media targeted at central carbon metabolites. Centroids represent the mean and concentration ellipses represent one standard deviation (level=0.68) of an estimated t-distribution based on the first two principal components.

B, PCA analysis of normal (blue; n=2), tumor (orange; n=3) and residual (green; n=3) populations based on genome wide DNA methylation data. The ellipses are confidence ellipses with 0.99 normal probability and the centroids represent the geometric center of the ellipse. This ellipse type was chosen because of the low number of replicates.

Data information: **A**-**B**, Number of replicates corresponds to different animals.

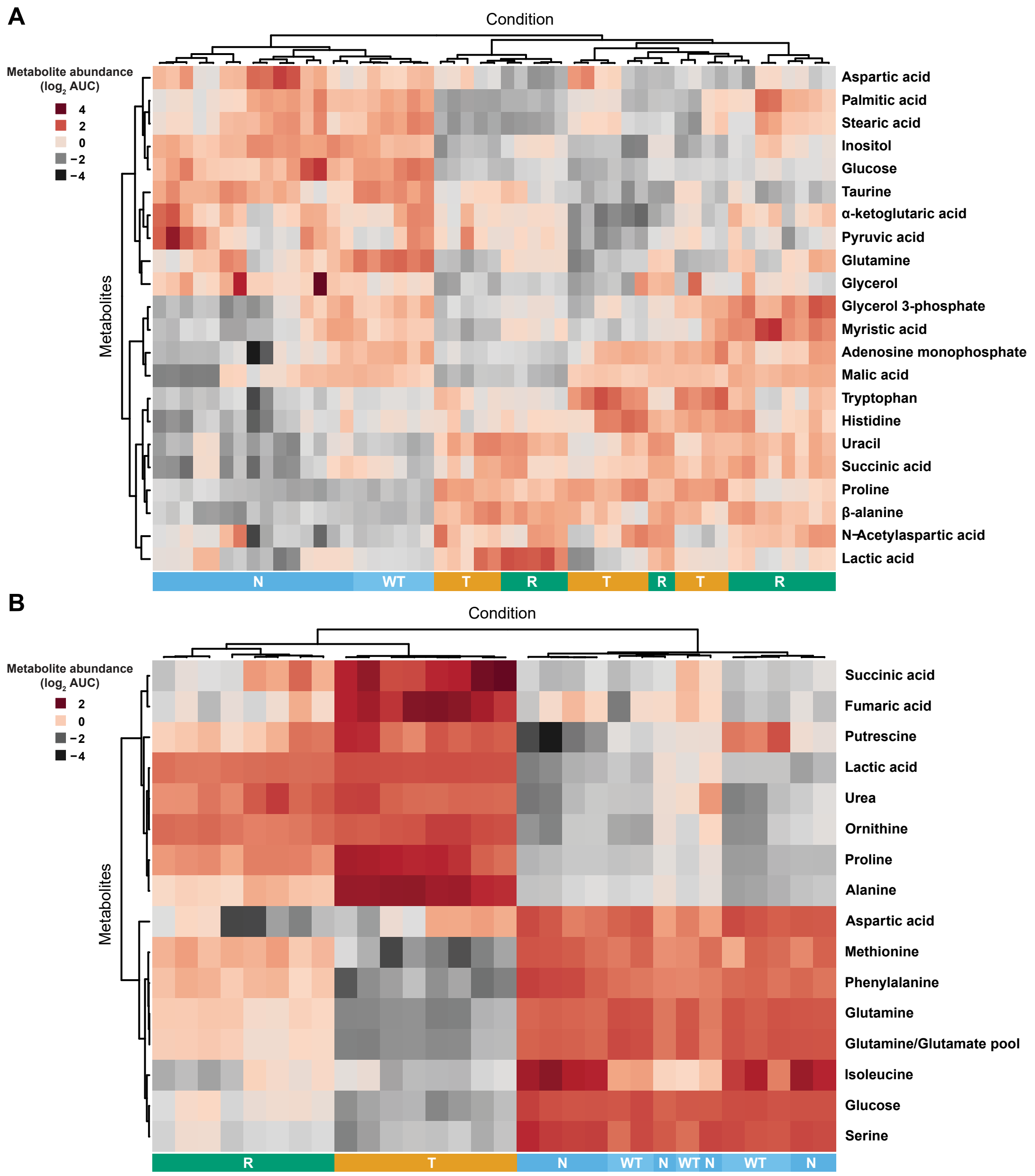

Appendix Figure S5: Analysis of measured intra- and extracellular metabolites demonstrates similarities between residual and tumor cells regarding their central carbon metabolism.

A-B, Heat maps representing the significantly altered metabolites in **(A)**, intracellular samples and **(B)**, extracellular spent growth media of residual (R, n=4) and tumor cells (T, n=4) compared to normal (N, n=4; WT n=2). The results are based on metabolomics analyses targeted at central carbon metabolites. The hierarchical clustering of the samples and the metabolites was based on Pearson's correlation using the complete linkage method. Statistics were calculated using the limma package (Ritchie, Phipson et al., 2015) in R with the significance threshold corresponding to a Benjamini-Hochberg adjusted *p*-value ≤ 0.05 (residual or tumor compared to normal). Values represent metabolite abundance levels as quantified by the area under the curve (AUC) of the marker fragment ions/transitions for each metabolite (log_2 scale). Number of replicates corresponds to different animals.

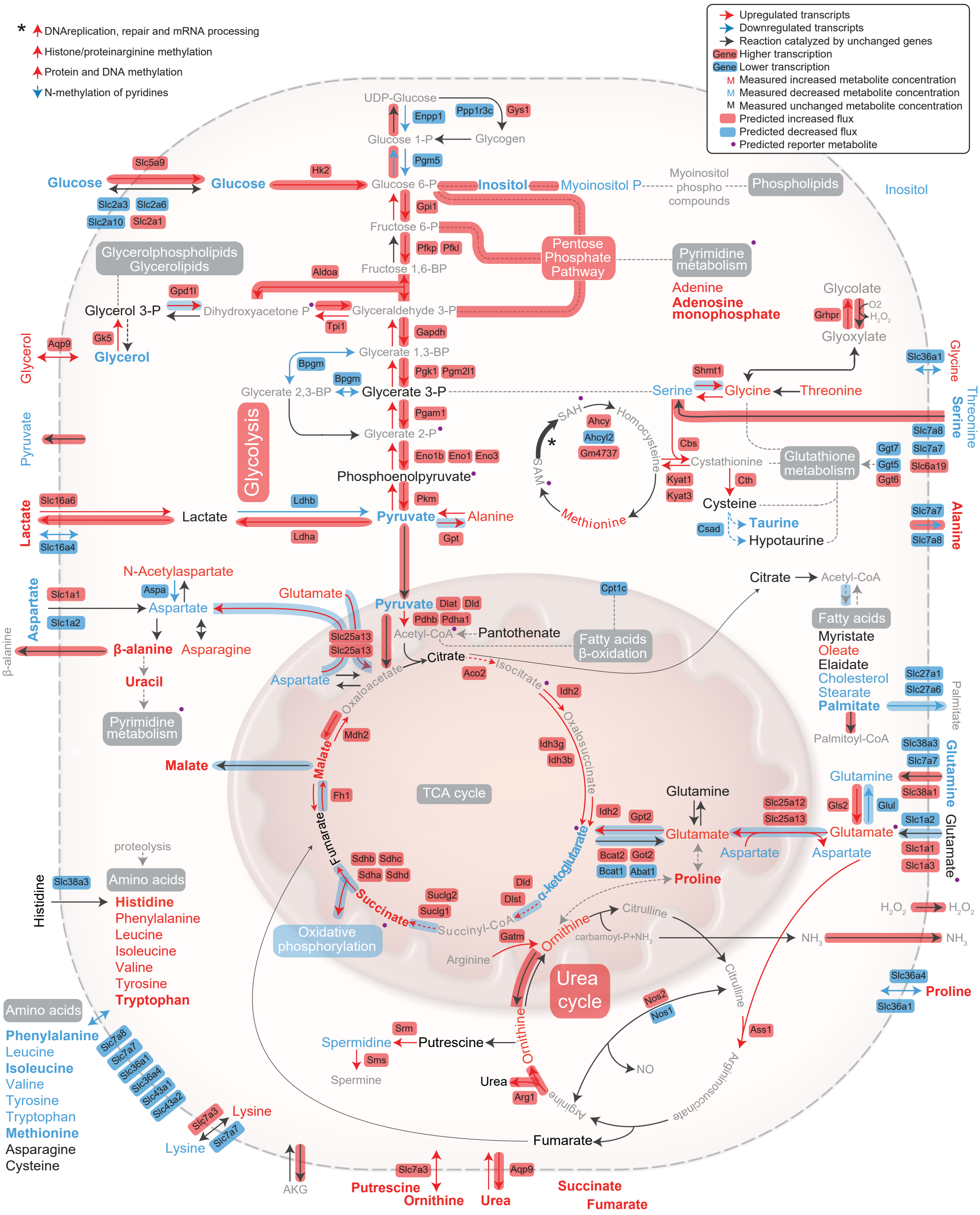

Appendix Figure S6: Global overview of altered metabolic pathways in tumor cells compared to normal in an extended representation.

A selection of genes with significantly altered expression (Bonferroni adjusted *p*-value < 0.1; normal n=8, tumor and residual n=4), targeted metabolites with significantly altered levels (Benjamini-Hochberg adjusted *p*-value ≤ 0.05; n=8; WT n=2) and significant reporter metabolites (top 5% with *p*-value < 0.1) of core metabolic processes are presented. Metabolites with an additional $\log_2$fold change (tumor compared to normal) ≥ 1 or ≤ -1 are highlighted in bold. A two-sided Wald test with a Negative Binomial GLM (Love et al., 2014) was used as test statistics for gene expression. For metabolite levels,

statistics were calculated using the limma package (Ritchie et al., 2015) in R with the significance threshold corresponding to a Benjamini-Hochberg adjusted *p*-value ≤ 0.05 (tumor compared to normal). For the reporter metabolites a gene set enrichment analysis was performed from a theoretical null- distribution using the reporter method (Varemo, Nielsen et al., 2013). Metabolite-gene sets were derived from a genome wide human metabolic model (HMR2), with genes mapped to mouse orthologs (Mardinoglu, Agren et al., 2014). Flux balance analysis predicted metabolic fluxes, which are altered in the corresponding pathways are overlaid. Significantly altered gene expressions and metabolites were used to inform the predictions. Number of replicates corresponds to different animals.

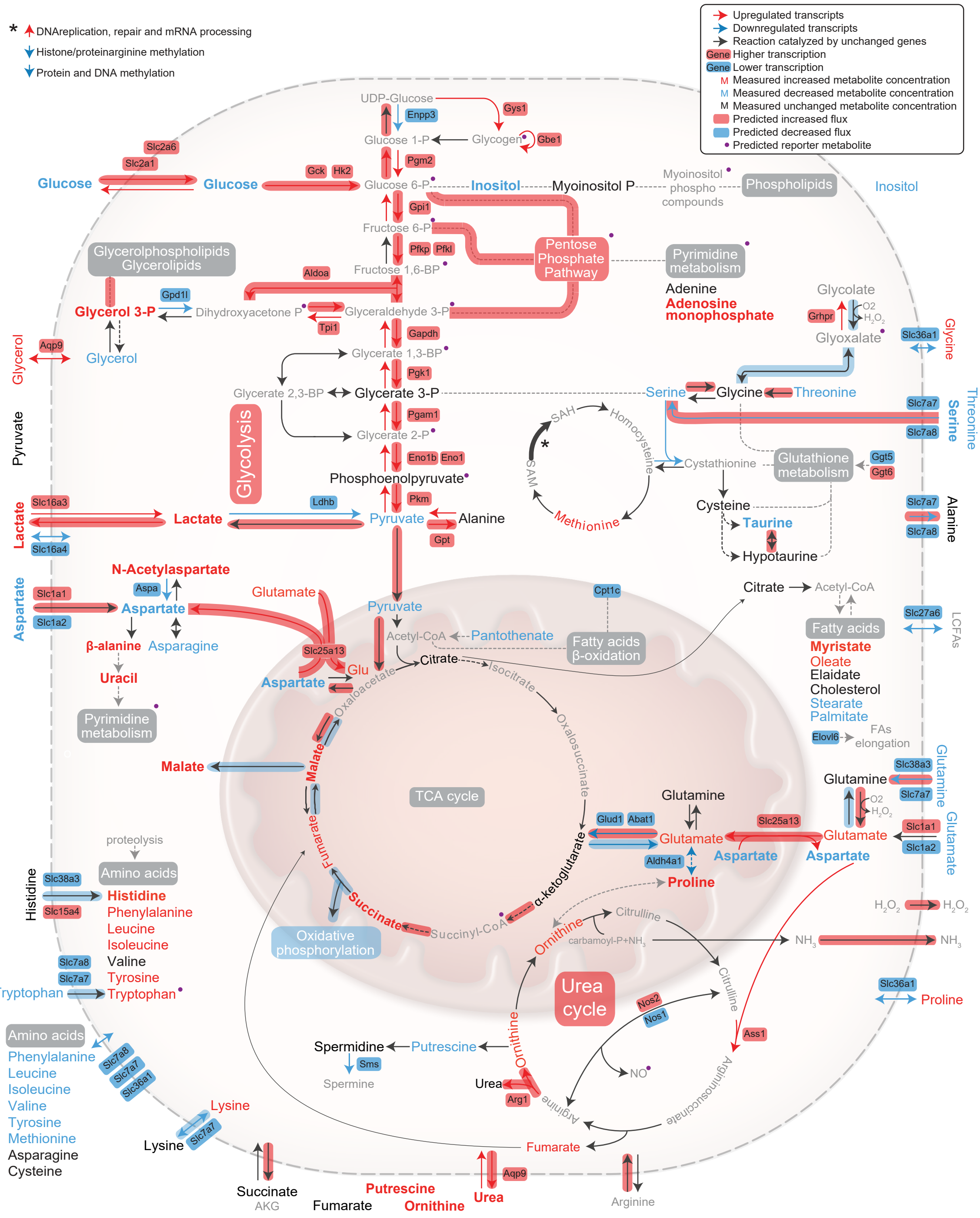

Appendix Figure S7: Global overview of altered metabolic pathways in residual cells compared to normal in an extended representation.

A selection of genes with significantly altered expression (Bonferroni adjusted *p*-value < 0.1; normal n=8, tumor and residual n=4), targeted metabolites with significantly altered levels (Benjamini-Hochberg adjusted *p*-value ≤ 0.05; n=8; WT n=2) and significant reporter metabolites (top 5% with *p*-value < 0.1) of core metabolic processes are presented. Metabolites with an additional $\log_2$fold change (residual compared to normal) ≥ 1 or ≤ -1 are highlighted in bold. A two-sided Wald test with a Negative Binomial GLM (Love et al., 2014) was used as test statistics for gene expression. For metabolite levels,

statistics were calculated using the limma package (Ritchie et al., 2015) in R with the significance threshold corresponding to a Benjamini-Hochberg adjusted *p*-value ≤ 0.05 (tumor compared to normal). For the reporter metabolites a gene set enrichment analysis was performed from a theoretical null-distribution using the reporter method (Varemo et al., 2013). Metabolite-gene sets were derived from a genome wide human metabolic model (HMR2), with genes mapped to mouse orthologs (Mardinoglu et al., 2014). Flux balance analysis predicted metabolic fluxes, which are altered in the corresponding pathways are overlaid. Significantly altered gene expressions and metabolites were used to inform the predictions. Number of replicates corresponds to different animals.

A

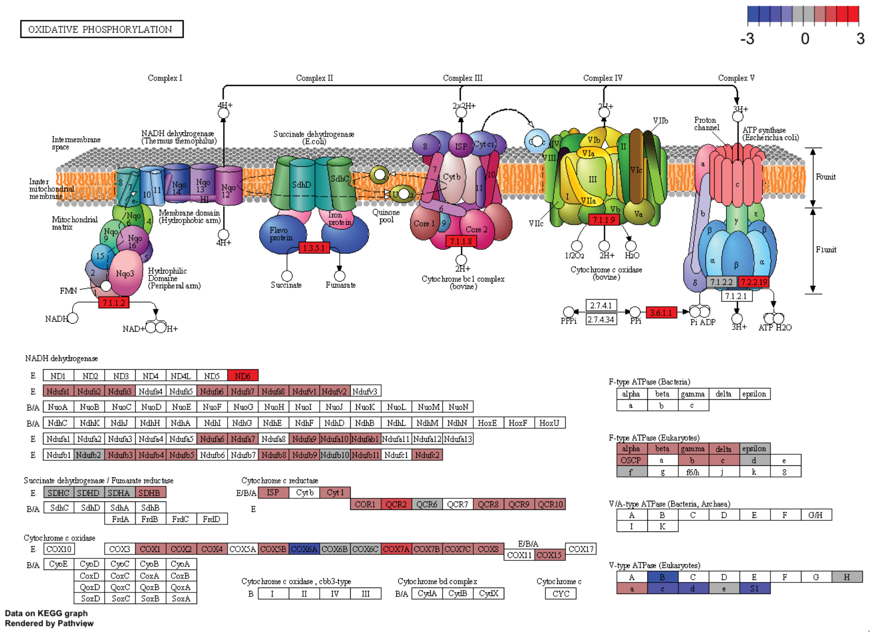

B

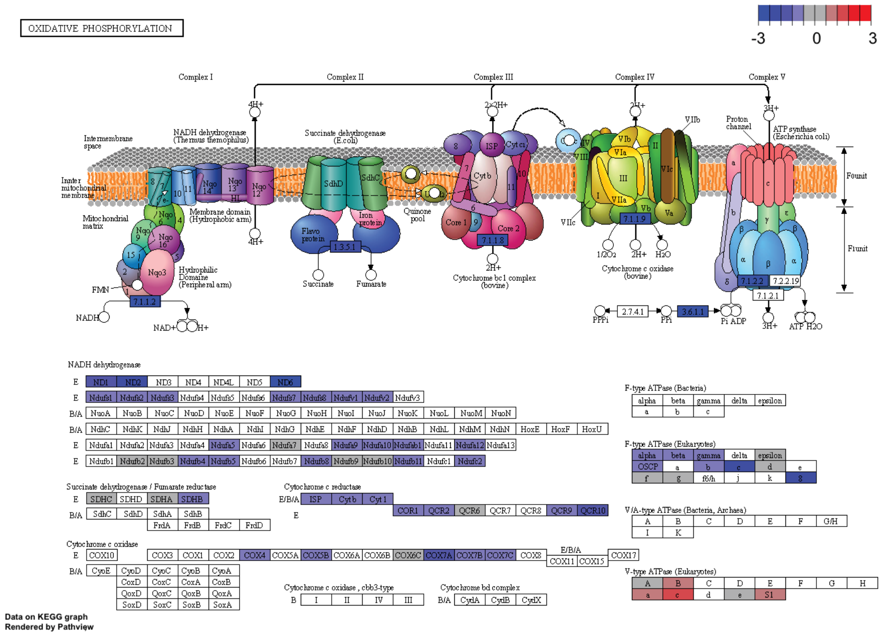

Appendix Figure S8: Transcript expression of genes from the Oxidative Phosphorylation (OXPHOS) pathway in tumor and residual cells.

A-B, Differentially expressed genes (two-sided Wald test, Bonferroni adjusted *p*-value < 0.1) of the **(A)**, tumor and **(B)**, residual cell populations in comparison to normal mapped on the KEGG pathway of Oxidative Phosphorylation (mmu00190). The coloring represents the $\log_2$fold with blue for down- and red for upregulated genes. Number of replicates (normal n=8, tumor and residual n=4) corresponds to different animals.

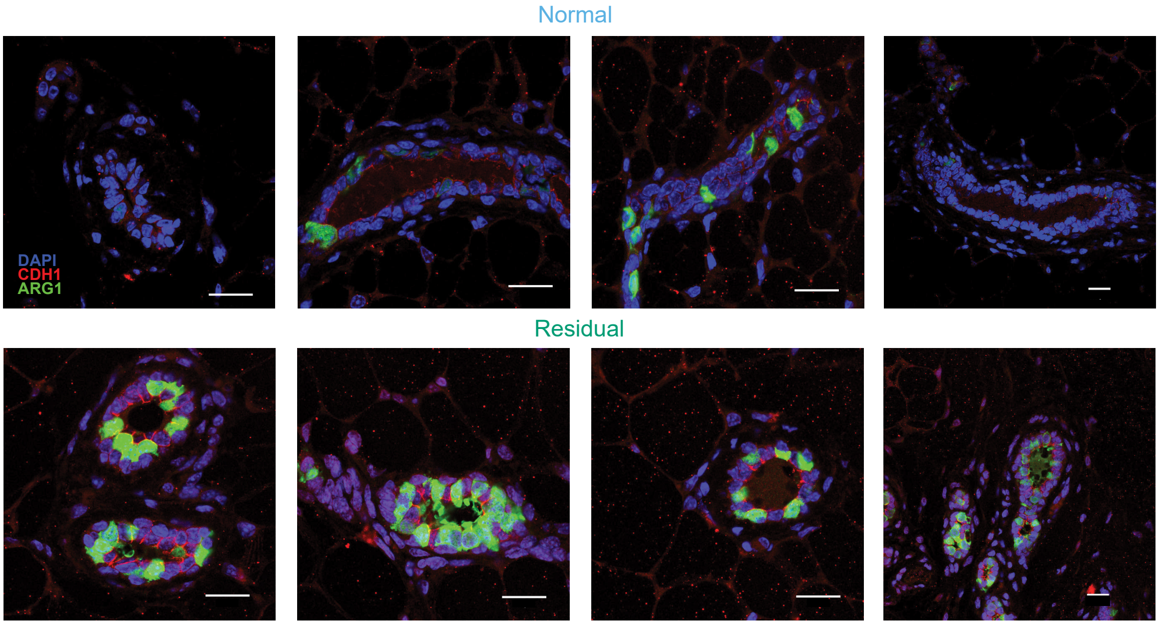

Appendix Figure S9: Immunofluorescence staining on mouse mammary tissue sections shows higher expression of Arginase1 in residual cells of regressed tissue as compared to normal cells of healthy tissue.
Representative images from staining of healthy (top) and residual ducts (bottom). Scale bar: 20 µm. Immunofluorescence: DAPI (blue), CDH1 (red), ARG1 (green).

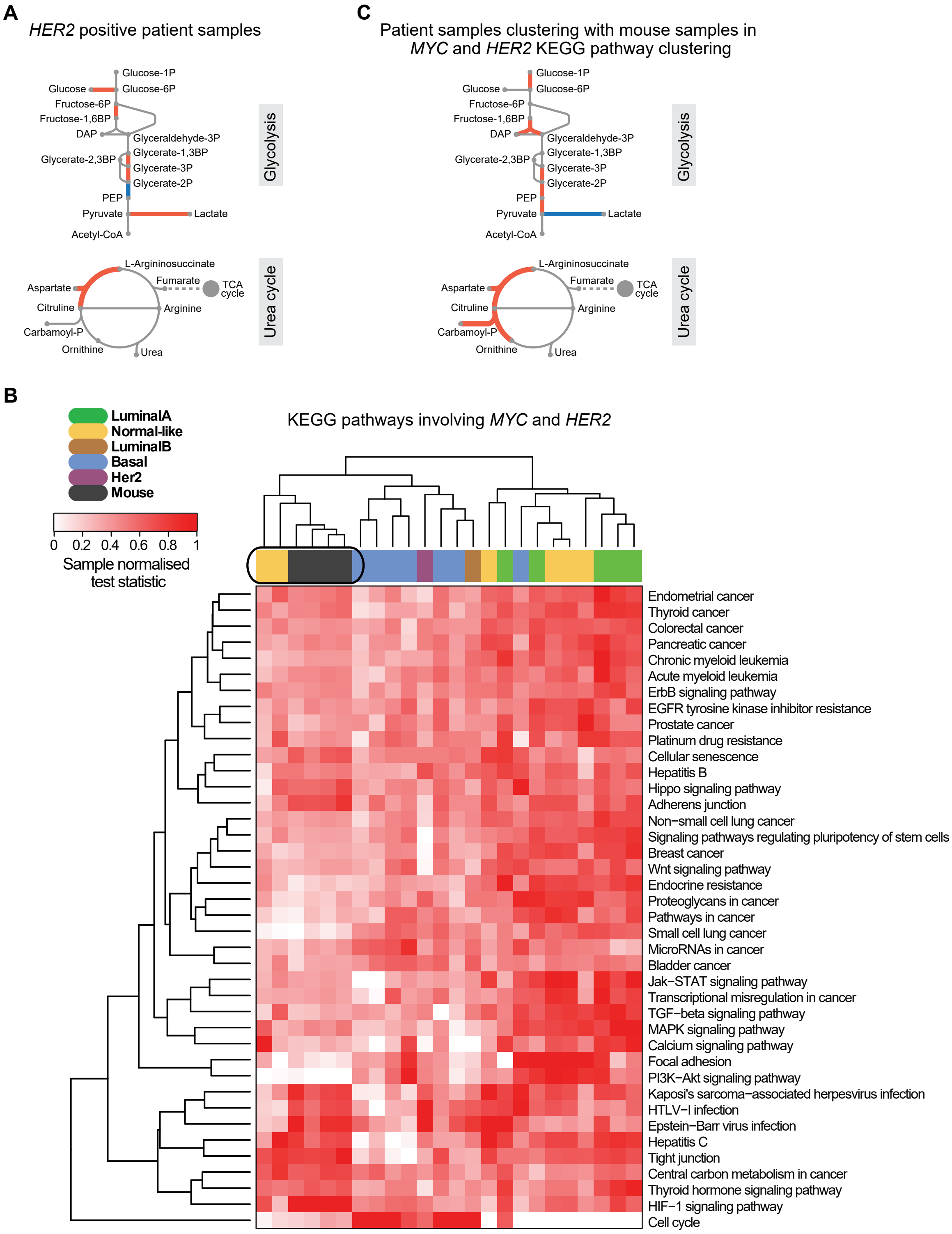

Appendix Figure S10: Extended validation of transcriptional changes in patient datasets.

A, Metabolic reactions of glycolysis and urea cycle, which are catalyzed by enzymes with differential expression (p-value < 0.05), are highlighted in red. An empirical Bayes moderated t-statistics was computed from a gene wise linear model fit with generalized least squares (Ritchie et al., 2015), comparing *HER2* positive classified patients (n=3 samples) with healthy breast tissue (n=10 samples). Differential expression from mouse *in vitro* transcriptome data of residual versus normal samples are depicted in Fig. 3G.

B, Joint clustering of sample-wise normalized individual pathway enrichment test statistics (unpaired one-side two-sample t-test) of mouse model (RNA-seq; normal, n=8; residual n=4) and patient samples (microarray; healthy, n=10, regressed n=20). Clustering is based on all genes of human KEGG pathways (or their mouse orthologs) that involve *HER2* or *MYC* (Methods). Hierarchical clustering with the complete linkage method and the Euclidean distance as a distance metric was used for clustering. For the patient comparison, two independent datasets, one from healthy breast tissue (GSE65194) (Maire et al., 2013, Maubant, Tesson et al., 2015) and one from patient tissues after neoadjuvant treatment (GSE32072) (Gonzalez-Angulo et al., 2012), were merged.
C, Metabolic reactions of glycolysis and urea cycle, which are catalyzed by enzymes with differential expression (p-value < 0.05), are highlighted in red. An empirical Bayes moderated t-statistics was computed from a gene wise linear model fit with generalized least squares (Ritchie et al., 2015), comparing the treated patients (n=4 samples), which are clustering closely with the mouse samples (encircled in **(B)**) with healthy breast tissue (n=10 samples). Differential expression from mouse *in vitro* transcriptome data of residual versus normal samples are depicted in Fig. 3G.
Data information: **A**-**C**, Number of replicates corresponds to different animals.

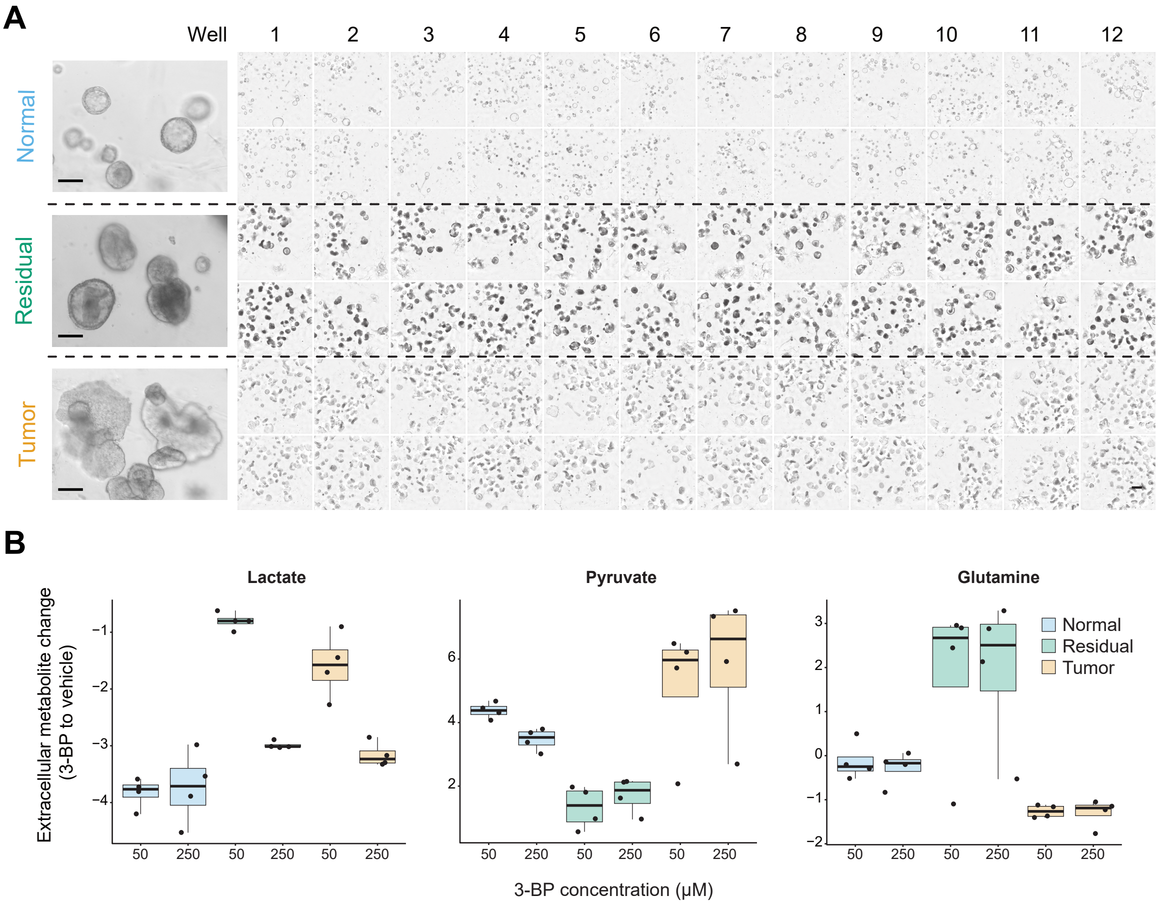

Appendix Figure S11: Treatment with 3-bromopyruvate functionally validates an altered glycolysis in the residual cells.

A, Representative bright field images in transmission showing 12 columns/6 rows of a 96-well plate containing normal (2 top rows), residual (2 middle rows) and tumor (2 bottom rows) structures before treatment with 3-BP; left panels representative enlarged images; scale bar 100 µm (left panel) and 500 µm (right panel).

B, Alteration of extracellular metabolite abundance levels in all three populations after treatment with 3-BP (n=4 each). The values represent the metabolite abundance ratio of 3-BP treated to untreated cells. Box plots: midline, median; box, 25–75th percentile; whisker, minimum to maximum. Number of replicates corresponds to different animals.

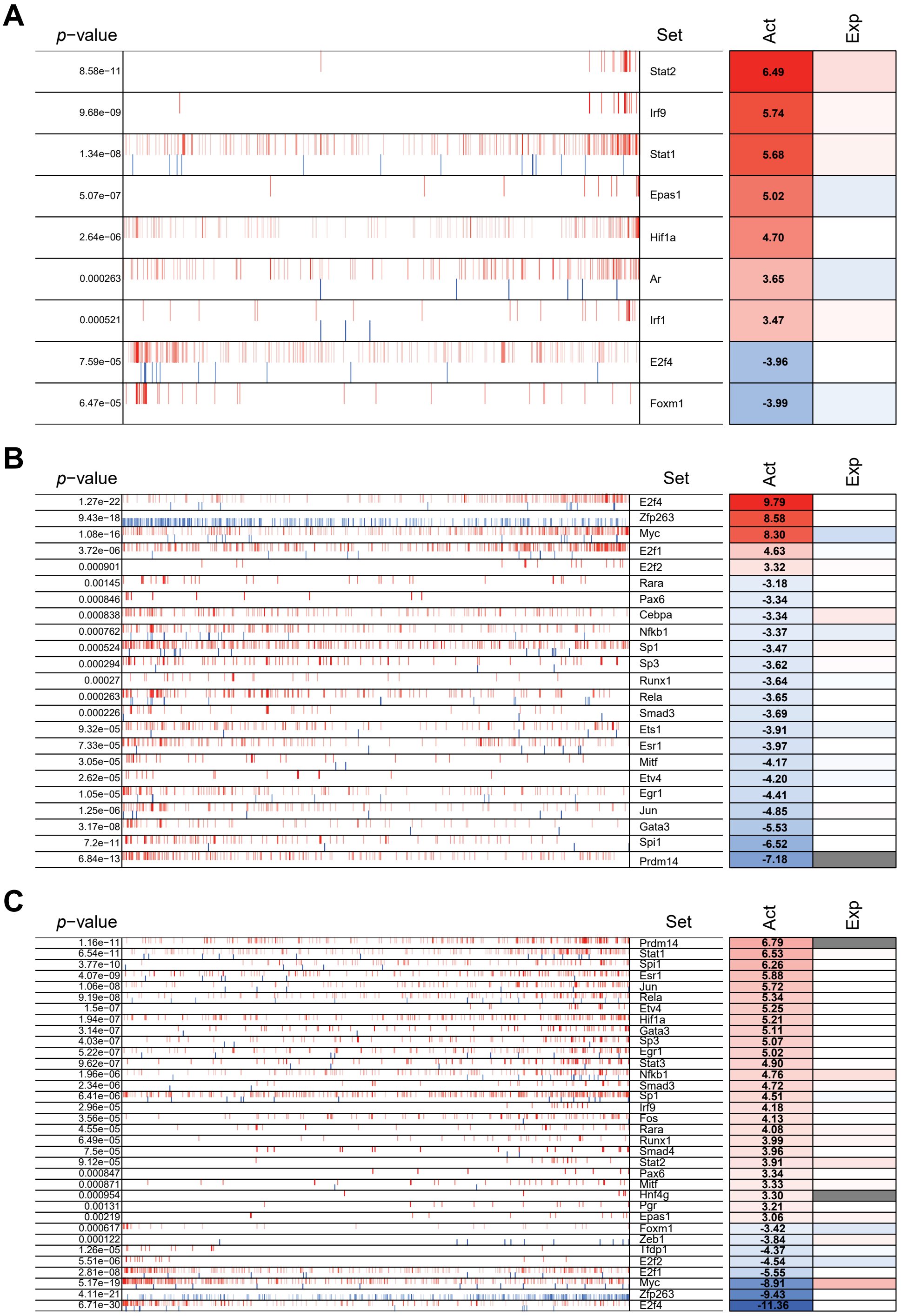

Appendix Figure S12: Transcription factor activity analysis reveals a distinct HIF1α signaling activity in residual cells.

A-**C**, Transcription factor (TF) activities were assessed with a footprinting based enrichment method using the differential gene expression of TF targets as an estimate for TF activity. Estimated activities (Act) are shown for significant TFs (Benjamini-Hochberg adjusted $p \leq 0.01$) comparing (**A**), residual versus normal (**B**), tumor versus normal and tumor versus normal and (**C**), residual versus tumor. Differentially expressed genes between the respective populations (two-sided Wald test, Bonferroni adjusted *p*-value < 0.1) and mouse transcription factor regulons (regulated target genes) from the bioconductor package “dorothea” with confidence levels “A” and “B” (Holland, Szalai et

al., 2020) were used as input for the msViper algorithm to calculate the activity (Alvarez, Shen et al., 2016). The bars in middle plot represent the whole regulon of the respective transcription factor with genes depicted in red being transcriptionally activated and in blue being transcriptionally inhibited by the respective transcription factor. The coloring on the estimated activity (Act) and expected activity under the null hypothesis (Exp) represents the TF activity strength with red for more and blue for less active TFs. The number of replicates in the transcriptomics data (normal n=8, tumor and residual n=4) corresponds to different animals.

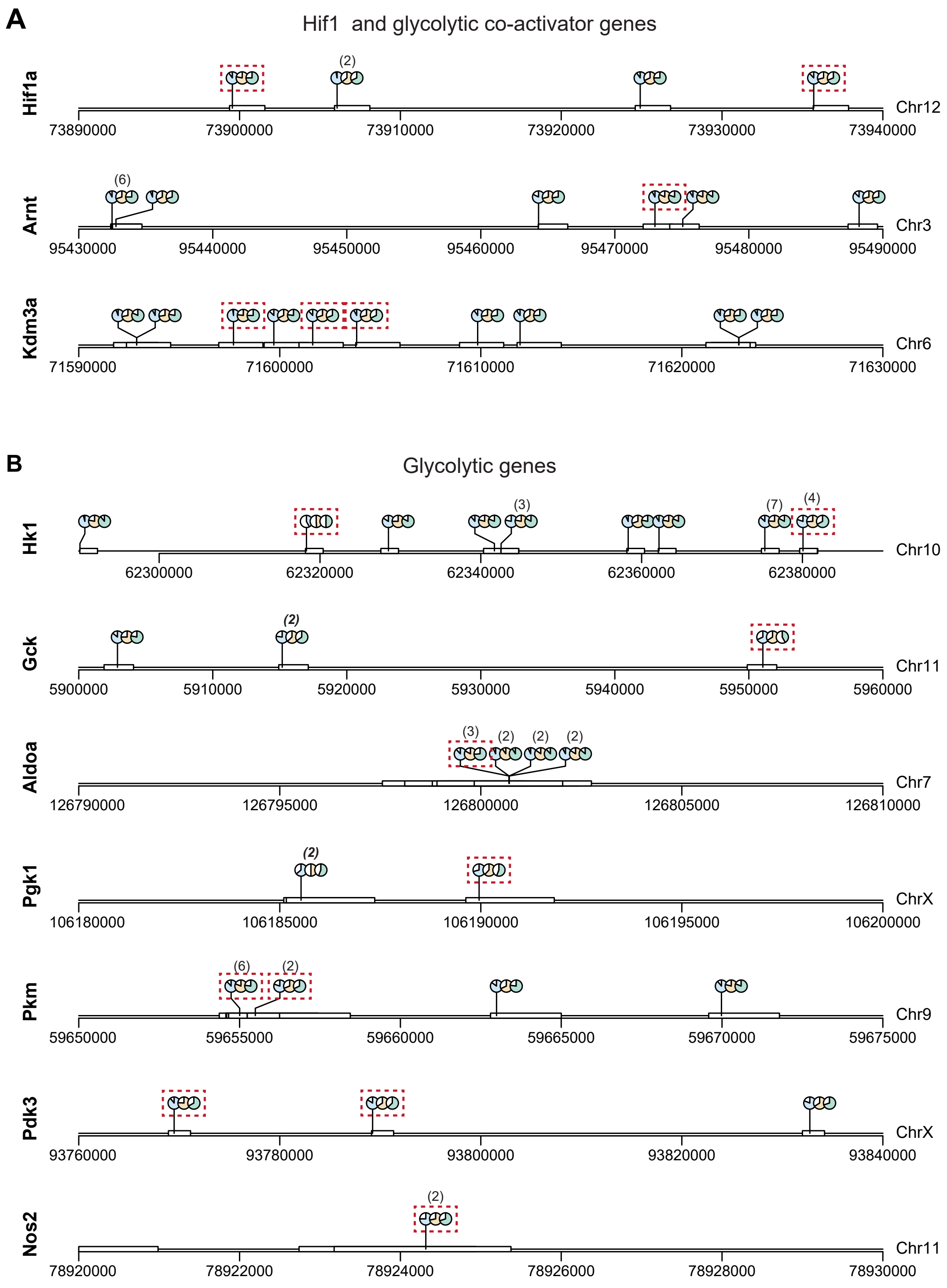

Appendix Figure S13: DNA methylation analysis of promoter regions of HIF1, HIF1 glycolytic co-activator genes and selected HIF1α target genes shows a consistent and isoform specific demethylation in tumor and residual cell populations.
Methylation percentages of CpG sites (as measured by bisulfide sequencing) are depicted within selected gene promoter regions for the normal (blue), tumor (yellow) and residual (green) cell population. The promoter regions (2000bp upstream and 200bp down stream of transcription start site) are shown for each gene's isoform individually as rectangular bars at their genomic location. Within each promoter region, the methylation

percentages of all CpG site were averaged. Identical isoform methylation percentages in all three populations of different promoters are only shown once with the number in brackets above the depiction indicating the number of identical iso forms. Only CpG sites with a minimum of 5 aligned reads in every sample were quantified. The number of replicates (normal n=2, tumor and residual n=3) corresponds to different animals.

Appendix Table S1: GO terms of significantly downregulated genes. Significant gene ontology terms (*p*-value < 0.001) from the gene ontology biological processes enriched for downregulated differentially expressed genes (Bonferroni adjusted *p*-value < 0.1) in the comparison residual vs. normal. A one-sided Fisher's exact test was used as test statistics. Number of replicates (normal n=8, tumor and residual n=4) corresponds to different animals.

GO Term ID	Description	Number of significant terms
GO:0006260	DNA replication	3
GO:0006928	movement of cell or subcellular component	1
GO:0007017	microtubule-based process	1
GO:0007049	cell cycle	1
GO:0007059	chromosome segregation	1
GO:0007154	cell communication	1
GO:0007155	cell adhesion	3
GO:0007188	adenylate cyclase-modulating G-protein coupled receptor signaling pathway	2
GO:0007267	cell-cell signaling	12
GO:0007610	behavior	1
GO:0008283	cell proliferation	1
GO:0009190	cyclic nucleotide biosynthetic process	16
GO:0009987	cellular process	1
GO:0019932	second-messenger-mediated signaling	1
GO:0022610	biological adhesion	1
GO:0023052	signaling	1
GO:0030198	extracellular matrix organization	1
GO:0030334	regulation of cell migration	8
GO:0032501	multicellular organismal process	1
GO:0032502	developmental process	1
GO:0042127	regulation of cell proliferation	1
GO:0043062	extracellular structure organization	1
GO:0044699	single-organism process	1
GO:0044707	single-multicellular organism process	37
GO:0044708	single-organism behavior	1
GO:0044763	single-organism cellular process	1
GO:0044767	single-organism developmental process	12
GO:0048285	organelle fission	17
GO:0048518	positive regulation of biological process	1
GO:0048519	negative regulation of biological process	3
GO:0048523	negative regulation of cellular process	18
GO:0048583	regulation of response to stimulus	1
GO:0050896	response to stimulus	1
GO:0051301	cell division	1
GO:0051302	regulation of cell division	1
GO:0051303	establishment of chromosome localization	2
GO:0055086	nucleobase-containing small molecule metabolic process	1
GO:0065007	biological regulation	1
GO:0071467	cellular response to pH	1
GO:0071840	cellular component organization or biogenesis	1
GO:1903047	mitotic cell cycle process	36
GO:1905818	regulation of chromosome separation	2

Appendix Table S2: GO terms of significantly upregulated genes. Significant gene ontology terms (*p*-value < 0.001) from the gene ontology biological processes enriched for upregulated differentially expressed genes (Bonferroni adjusted *p*-value < 0.1) in the comparison residual vs. normal. A one-sided Fisher's exact test was used as test statistics. Number of replicates (normal n=8, tumor and residual n=4) corresponds to different animals.

GO Term ID	Description	Number of significant terms
GO:0001775	cell activation	1
GO:0001816	cytokine production	11
GO:0001906	cell killing	1
GO:0001909	leukocyte mediated cytotoxicity	2
GO:0002376	immune system process	1
GO:0002831	regulation of response to biotic stimulus	1
GO:0006482	protein demethylation	1
GO:0006915	apoptotic process	2
GO:0006928	movement of cell or subcellular component	1
GO:0006952	defense response	8
GO:0006955	immune response	69
GO:0007154	cell communication	1
GO:0007166	cell surface receptor signaling pathway	10
GO:0008214	protein dealkylation	1
GO:0008219	cell death	1
GO:0008283	cell proliferation	1
GO:0009132	nucleoside diphosphate metabolic process	17
GO:0009607	response to biotic stimulus	1
GO:0018108	peptidyl-tyrosine phosphorylation	6
GO:0022610	biological adhesion	1
GO:0023052	signaling	1
GO:0030029	actin filament-based process	1
GO:0030036	actin cytoskeleton organization	2
GO:0032501	multicellular organismal process	1
GO:0032502	developmental process	1
GO:0032879	regulation of localization	1
GO:0034097	response to cytokine	1
GO:0034330	cell junction organization	1
GO:0040011	locomotion	1
GO:0040012	regulation of locomotion	1
GO:0042107	cytokine metabolic process	1
GO:0042127	regulation of cell proliferation	2
GO:0044699	single-organism process	1
GO:0044763	single-organism cellular process	1
GO:0045216	cell-cell junction organization	5
GO:0048518	positive regulation of biological process	1
GO:0048519	negative regulation of biological process	3
GO:0048522	positive regulation of cellular process	5
GO:0048523	negative regulation of cellular process	7
GO:0048856	anatomical structure development	29
GO:0050790	regulation of catalytic activity	21
GO:0050896	response to stimulus	1
GO:0051270	regulation of cellular component movement	4
GO:0051674	localization of cell	1
GO:0065009	regulation of molecular function	1
GO:0070988	demethylation	1
GO:0072524	pyridine-containing compound metabolic process	1
GO:0090002	establishment of protein localization to plasma membrane	5
GO:0098609	cell-cell adhesion	14

Appendix Table S3: Footprinting based transcription factor enrichment of the residual cell population in comparison to normal and tumor.

The transcription factor (TF) enrichment was assessed by using the gene expression of TF target genes as an input for the enrichment calculation (footprinting approach). Genome-wide rlog transformed gene counts and mouse transcription factor regulons (regulated target genes) from the bioconductor package "dorothea" with confidence levels "A" and "B" (Holland et al., 2020) were used as input for the viper algorithm (Alvarez et al., 2016) to calculate the enrichment. Estimated activities are shown for significant TFs (Benjamini-Hochberg-adjusted $p \leq 0.01$). The number of replicates in the transcriptomics data (normal n=8, tumor and residual n=4) corresponds to different animals.

TF activities of the residual cell population

TF	T-Value	*P*-Value	FDR
Hif1a	8.17	1.07E-06	0.000129
Epas1	7.5	2.86E-06	0.000173
Zeb1	-6.95	6.75E-06	0.000272
Fosl2	6.07	2.91E-05	0.000704
Irf9	6.18	2.41E-05	0.000704
Stat1	5.52	7.50E-05	0.001513
Stat2	5.27	0.000118	0.001845
Stat6	5.26	0.000122	0.001845
Foxm1	-5.05	0.000176	0.002366
Mycn	-4.65	0.000373	0.004513
Esr1	4.23	0.000835	0.009185
