# Supplementary material for: Metabolic memory underlying minimal residual disease in breast cancer
Source: Mol Syst Biol. 2021 Oct 25;17(10):e10141. doi: 10.15252/msb.202010141 (PMC8543468; doi:10.15252/msb.202010141)
Supplement: Supplementary file 3 [file MSB-17-e10141-s002.pdf]

Metabolic memory underlying minimal residual disease in breast cancer

Ksenija Radic Shechter, Eleni Kafkia, Katharina Zirngibl, Sylwia Gawrzak, Ashna Alladin, Daniel Machado, Christian Lüchtenborg, Daniel Sevin, Britta Bruegger, Kiran Patil, and Martin Jechlinger
DOI: 10.15252/msb.202010141

Corresponding author(s): Kiran Patil (kp533@cam.ac.uk) , Martin Jechlinger (martin.jechlinger@embl.de)

Review Timeline:

Submission Date:	24th Nov 20
Editorial Decision:	25th Nov 20
Revision Received:	3rd Aug 21
Editorial Decision:	13th Aug 21
Revision Received:	23rd Sep 21
Accepted:	29th Sep 21

Editor: Maria Polychronidou

Transaction Report:

(Note: With the exception of the correction of typographical or spelling errors that could be a source of ambiguity, letters and reports are not edited. Depending on transfer agreements, referee reports obtained elsewhere may or may not be included in this compilation. Referee reports are anonymous unless the Referee chooses to sign their reports.)

1st Editorial Decision **25th Nov 2020**

Thank you for transferring your manuscript to Molecular Systems Biology. I have now read your study and the reviewers' comments. I think that the study sounds interesting and we would be happy to consider it for Molecular Systems Biology. I would therefore invite you to perform a revision to address the issues raised by the referees. I will then consult with a new reviewer or an Editorial Advisory Board member on whether the revised study seems well suited for MSB.

I think that the reviewers from EMBO Molecular Medicine have provided thorough evaluations and it is therefore not necessary to review the study from scratch. However, as they reviewed having in mind the scope and more translational requirements of EMBO Molecular Medicine (e.g. they make no comments on the metabolic modelling, data integration etc.) I would like to have a referee or EAB member providing comments specifically for Molecular Systems Biology and placing more emphasis on the systems-level aspects of the work. I hope this sounds reasonable.

In line with what Kiran and I discussed in the call, I feel that adding 13C flux analyses, including comparisons of residual cells to tumor cells and clarifying the novelty compared to the Havas et al, 2017 study up-front would be important. As a more minor comment, I noticed that the manuscript is currently presented in a rather condensed report-like format. In principle we do not have specific requirements regarding formatting. However, I think that the manuscript would benefit from being organised into Introduction, Results and Discussion sections, with the Results being split into sub-sections so that it is easier for the reviewer(s) to access the main findings.

REFEREE REPORTS

Referee #1 Review
Received: 5th Nov 20

Remarks for Author:
The authors studied MRD in a breast cancer organoid model and compared it to mouse models and to data of human patients at the transcriptomic level. They find reminiscent metabolic changes in the remaining cells involving enhanced glycolysis and urea cycle abnormalities that cluster with similar changes in the tumor. They suggest that residual cells carry a metabolic and epigenetic memory of the primary tumor.

My main concern is regarding the paper published by this group in JCI 2017. I think the major novelties of the system as demonstrated here, have been demonstrated already in the previous paper. Also, there is a discrepancy between the two papers regarding lipid metabolism- in the 2017 paper, they find distinctive alterations in lipid metabolism between the residual cells in the organoids and the primary tumors, while here they emphasize the similarities in lipid metabolism between the organoids and tumors and refer to it as a metabolic memory.
In addition, some of the data analyses are unorthodox- for example, having mouse and human transcriptome cluster together. Similarly for the statistics, for example they use T test in 4a where Anova is more appropriate. And most graphs miss the * making hard to evaluate the findings for significance.
Specific comments-
Metabolic aspects- crucial data is missing regarding OXPHOS activity to the point that it is absent from the metabolic map in Fig.2. It is hence hard to evaluate potential changes in glycolysis without knowing if there are any changes in OXPHOS. Also, the authors suggest that the enhanced glycolysis is a metabolic memory reminiscing from the primary tumor and yet only the residual cells and not the tumors, respond to the glycolysis inhibitor, which seems to be contradictory.
As for the urea cycle, the authors likely confuse it with the arginine-citrulline cycle, which is the one present outside the liver. If they want to claim the urea cycle pathway is active in breast cancer cells, they need to show enzymatic expression of all urea cycle enzymes and the levels of urea cycle intermediate metabolites as arginine, carbamoylphosphate, argininosuccinate etc.

The ones they are showing- aspartate, fumarate, proline and putrescine, are not urea cycle direct intermediates. The high levels of urea and ornithine they find support high ARG activity as indeed they further demonstrate but does not imply urea cycle activation as a pathway. The authors also find high NOS activity, which usually competes over arginine with ARG. It is hence important to show whether the main metabolic outcome of these metabolic changes relate to changes in NO levels or in polyamines. In addition, there are 3 NOS isoforms, which one's activity did they measure?
Finally, high arginase and enhanced glycolysis are established hallmarks of aggressive breast cancer. Hence it might not be surprising to see them in the residual cells.

Minor comments-
S5- hard to compare since they show different metabolites for the intra and extracellular fractions.
Fig. 4b- unclear what is being demonstrated. Are these representative images for 4a? The data regarding the metabolic effects on the epigenome are only speculative.

Referee #2 Review
Received: 8th Nov 20

Remarks for Author:

Remarks to the Author:

In this manuscript, the authors performed the metabolomics, lipidomics and transcriptomics analysis of minimal residual disease (MRD) based on a tractable organoid system, and uncovered the elevated glycolysis and dysregulated urea cycle in residual organoids, which was also observed in a mouse model and in transcriptomic data from patients upon neo-adjuvant therapy. They further found a similarity in DNA methylation profiles between tumor and residual cells. Intervening glycolysis with a small molecule inhibitor could suppress the cell survival, suggesting a potential target for preventing breast tumor recurrence. This study offers the multi-omics data to study the metabolic alterations of MRD, however, there are a few issues to be clarified.

1. The authors claimed the residual organoids they established were referred to as "residual cells" which constitute the treatment-tolerant cancer cells. However, they did not confirm whether the residual organoids were treatment-tolerant or treatment-resistant. It would be necessary to establish residual organoids with treatment of high dose anti-cancer drugs and determine the metabolic alterations of the surviving residual cells upon drug treatment by multi-omics analysis.
2. In figure 1b and figure S2, the distinct transcriptomics profiling between residual cells and normal cells was not surprising. It is also important to perform the gene-set enrichment analysis comparing the residual and tumor cells to identify the difference between residual cells and tumor cells.
3. Like the issues in transcriptomics analysis, the authors should analyze the difference between residual cells and tumor cells based on the metabolomic and lipidomic data. This will contribute to identify new biomarkers and targeted therapeutic strategy for MRD.
4. The authors performed lipidomic analysis but they did not mention the specific alterations in lipids in the manuscript and figures. A more detailed description of the lipidomic profiling would be needed.
5. The glycolysis inhibitor 3-BP has been regarded as a potential anti-cancer drug. In figure 4a, the tumor cells stayed close to the baseline viability after exposure to 3-BP is not rational. In fact, we can observe a marked difference in morphology of the tumor cells with or without treatment of 3-BP in figure 4b.
6. To clarity the dynamic metabolism changes, the authors would be better to perform the metabolic flux assessed by stable isotope labeling.

Referee #3 Review
Received: 9th Nov 20

Comments on Novelty/Model System for Author:
In this manuscript, a novel concept of metabolic memory is proposed. The authors show that treatment-resistant cancer cells, despite having a quiescent phenotype, still have a metabolic profile similar to cancer cells. They claim that this specific metabolic features represent a vulnerability of the residual cells, notably to glucose deprivation.
This is very original data that could represent, if properly revised, an important finding in the field of cancer metabolism. In the present form, however, the data presented is not yet conclusive enough, and further experiments should be performed.

1. As a result of switching-on Myc expression, upon doxycycline treatment, cells were transformed and generated a tumor-like structure. Myc-expressing cells had a distinct gene expression signature, which was consistent with tumor formation. After the extinction of Myc expression, tumor cells regressed, but the gene expression profile (at least at a significant extent) was similar to the tumor cells. The question here is how the expression of these genes is still changed if the triggering factor, Myc, is absent? Which factors contribute to the sustained expression of these genes?

2. Lipids composition is shown to be different in tumor cells and in resistant cells, but these differences are not detailed, neither is explained the significance of the distinct lipid profiles.

3. Metabolic studies in the cells using Seahorse analysis could provide functional data to support the changes observed in metabolomics and lipidomics studies.

4. In the figure 3, the metabolic profile of residual cancer cells from tumors in mice is compared to normal cells, and it is shown that glycolysis and urea cycle pathways are increased in these cells. However, an important control, tumor cells, is missing in this experiment.

5. The authors compare the transcriptomics data of patients before and after neoadjuvant therapy and show a similar gene expression profile in residual cells. However, under the view of this reviewer, this comparison is not conclusive, because the authors cannot discriminate between residual cells and "remaining" cancer cells. Indeed, they should show that, similar as in the organoids results, the patient samples that they are analyzing also have deregulated cell cycle and cell proliferation markers as well as upregulated cytoskeletal and adhesion genes.

6.It is also shown that inhibition of glycolysis specifically killed residual cells. This treatment was not efficient, however, to kill tumor cells. This result is counterintuitive, since tumor cells have also increased glycolysis. How tumor cells overcome the inhibition of glycolysis? What alternative pathways are triggered in these cells, which are not active in the residual cells? A comparative analysis would provide some clues about the metabolic changes in residual cells that renders them more sensitive to glucose deprivation.

We thank all referees for their critical and diligent review, which helped us improve the manuscript substantially.
Point-by-point response to the reviewer's comments are provided below.

Referee #1 (Remarks for Author):

The authors studied MRD in a breast cancer organoid model and compared it to mouse models and to data of human patients at the transcriptomic level. They find reminiscent metabolic changes in the remaining cells involving enhanced glycolysis and urea cycle abnormalities that cluster with similar changes in the tumor. They suggest that residual cells carry a metabolic and epigenetic memory of the primary tumor.

My main concern is regarding the paper published by this group in JCI 2017. I think the major novelties of the system as demonstrated here, have been demonstrated already in the previous paper.

→ Our study has several novel aspects compared to the Havas et al., 2017 JCI paper (Havas et al.):

- **Three-way comparison**: We provide a comprehensive comparison of all the 3 states (normal, tumor, and residual). We characterize these states at the epigenetic, transcriptomic, and metabolic levels, and integrate the latter two to provide a holistic view of the metabolic pathophysiology. In contrast, this was neither the aim nor shown in the former work, that focused on deregulation between normal and residual cells. The multiomic analysis of the present study brought forward similarities between tumor and residual state that are representing common therapeutic targets and are not described in Havas et al. Notably, it would not have been possible to identify these targets in the Havas paper, due to the lack of a complete three-way comparison and also due to the lack of comprehensive metabolomics analysis.
- **Methodological advance:** The comprehensive three-way comparison of the present study is built upon novel methodological developments for 3D cultures.
- **Metabolic memory:** The concept of the metabolic memory in residual cells is a novel finding of the current study, and to our knowledge this concept has not been described in the field overall (as also pointed by two of the reviewers). The molecular basis for the memory was confirmed by epigenetic analysis. As such, the concept of metabolic memory could not have been concluded from Havas et al., which rather offered a coarse description based on transcriptome profiles using microarray analysis and compared only the normal and residual states.
- **Metabolic similarity between tumor and MRD states:** Although aggressive breast cancer tumors display deregulated glycolysis and urea metabolism, there is no description so far that the residual cells, which are highly phenotypically akin to normal cells and hence escape detection through conventional methods, retain such characteristics. In fact, this is one of the novel and surprising findings from our study. Even beyond the breast cancer case, increased glycolysis has rarely been, if at all, associated with non-proliferative state.
- **Novel therapeutic target:** While glycolytic phenotype was observed for both tumor and residual cells, only the residual cells showed vulnerability to the glycolysis inhibition. While this observation might appear as counterintuitive, it is a direct consequence of the metabolic memory. While the metabolic plasticity of the tumor state allows them to utilize alternative pathways, the residual cells become vulnerable due to their reliance on glycolysis in contrast to the starting normal state. We could thus selectively target the unwanted residual cells using a glycolytic inhibitor.

To make clear that we developed these advances and insights on the base of prior published work and model systems, we have now rephrased the introduction accordingly (lines 38-48). Further we have worked on the discussion to highlight some of the rationale outlined

Also, there is a discrepancy between the two papers regarding lipid metabolism- in the 2017 paper, they find distinctive alterations in lipid metabolism between the residual cells in the organoids and the primary tumors, while here they emphasize the similarities in lipid metabolism between the organoids and tumors and refer to it as a metabolic memory.

Regarding the comment on the perceived contradictory results on lipid metabolism between the current manuscript and Havas et al. 2017, we need to clarify an apparent misunderstanding:

- The Havas et al. paper mainly describes the state of residual disease in comparison with normal control (figures 2-7 in Havas et al.), which includes the differences in lipid metabolism (normal versus residual, no tumor state shown or analyzed in these figures). The similarity between the lipid

metabolism of tumor and residual disease can be appreciated in the FASN immune-staining in Figure 8 (patient material correlating the finding, again focusing on normal versus residual, also showing tumor) in Havas et al. The known/similar deregulated lipid metabolism in the breast tumor state is also pointed out in the discussion of the prior manuscript.

- The similarity of the lipidomic profiles of residual and tumor cells as compared to the normal state (as shown in the current study) does not mutually exclude a degree of distinction and the presence of alterations amongst the tumor and residual state (as stated in the previous study with regards to fatty acid oxidation (Figure 1c in Havas et al.)). The two studies employ different methodologies that tackle different aspects of lipid metabolism and hence are rather complementary and not contradicting. The Havas paper focused on fatty acid oxidation and offered a biochemical analysis for palmitate oxidation and staining for lipid droplets. The current study employed an unbiased characterization of lipid species through mass spectrometry.
- Finally, our concept of metabolic memory is not based on lipidomics data as reviewer suggests, but rather on integrative analysis of RNAseq, metabolomics, epigenetics, and metabolic modelling – all supported by phenotypic observations.

We refer to the "the close similarity between residual and tumor populations in agreement with our previous work" in the revised version (line 84-86).

In addition, some of the data analyses are unorthodox- for example, having mouse and human transcriptome cluster together.

→ This is a plus point of our study and demonstrates the effort we have taken to place our findings in the patient context. Such analysis is critical for any model system, and we believe that our approach sets up a good example for future studies.

Similarly for the statistics, for example they use T test in 4a where Anova is more appropriate. And most graphs miss the * making hard to evaluate the findings for significance.

→ The t-test is appropriate when comparing two groups as is the case in point. The statistical criteria are mentioned in the figure legend and the relevant supplementary tables. We have included all significance * markings (and/or p-values) in the figures, The * markings are not included to avoid overcrowding in the figures.

Specific comments-
Metabolic aspects- crucial data is missing regarding OXPHOS activity to the point that it is absent from the metabolic map in Fig.2. It is hence hard to evaluate potential changes in glycolysis without knowing if there are any changes in OXPHOS.

→ Oxphos is now included in Figure 2 and supplementary figures 6, 7 and 8. The results for OXPHOS are well supported by the integrative metabolic modelling that accounts for a) changes observed at transcription level, b) changes in metabolite levels, and c) mass balance constraints on the system.
The metabolic modeling predicts OXPHOS to be lower/less active in comparison to glycolysis in both, tumor and residual cells.

In addition, we have now included new data showing, in agreement with the integrative modelling, that the higher glucose metabolism to lactate in the residual population, as compared to normal cells, is further validated by the increased labeling of lactate following supplementation with [U-13C]glucose.

Also, the authors suggest that the enhanced glycolysis is a metabolic memory reminiscing from the primary tumor and yet only the residual cells and not the tumors, respond to the glycolysis inhibitor, which seems to be contradictory.

→ While the glycolytic phenotype was observed for both tumor and residual cells, only the residual cells showed vulnerability to the glycolysis inhibition. While this observation might appear as counterintuitive, it is a direct consequence of the metabolic memory. While the metabolic plasticity of the tumor state allows them to utilize alternative pathways, the residual cells become vulnerable due to their reliance on glycolysis in contrast to the starting normal state. The flux through oxidative phosphorylation (OXPHOS) in relation to glycolysis was predicted by the model to be lower in both residual and tumor cells than in the normal cells. Yet, transcript levels of OXPHOS associated genes indicated that the tumor cells harbored higher capacity for OXPHOS than utilized at the flux level (Figure S8). The excess capacity of tumors in central metabolic pathways has been noted before as a buffer in the face of perturbations (e.g., Park et al., Nat Chem Biol, 2019).

We now further extended and renewed the paragraph discussing the potential roots of higher glycolytic dependence of residual cells as compared to tumor cells (line 171-173). We cite and present data towards (1) lower OXPHOS capacity, (2) less anti-apoptotic signal pathway acitivity and (3) reduced metabolic flexibility in residual cells in comparison to tumor cells.

As for the urea cycle, the authors likely confuse it with the arginine-citrulline cycle, which is the one present outside the liver. If they want to claim the urea cycle pathway is active in breast cancer cells, they need to show enzymatic expression of all urea cycle enzymes and the levels of urea cycle intermediate metabolites as arginine, carbamoylphosphate, argininosuccinate etc. The ones they are showing- aspartate, fumarate, proline and putrescine, are not urea cycle direct intermediates. The high levels of urea and ornithine they find support high ARG activity as indeed they further demonstrate but does not imply urea cycle activation as a pathway. The authors also find high NOS activity, which usually competes over arginine with ARG. It is hence important to show whether the main metabolic outcome of these metabolic changes relate to changes in NO levels or in polyamines. In addition, there are 3 NOS isoforms, which one's activity did they measure?
Finally, high arginase and enhanced glycolysis are established hallmarks of aggressive breast cancer. Hence it might not be surprising to see them in the residual cells.

→ While the complete urea cycle takes place in the liver, individual enzymatic components of the urea cycle are also expressed outside the liver, in different tissues (Lee, Adler et al., 2018). In this manuscript, we refer to these specific enzymatic steps, and we re-phrased accordingly to avoid the confusion.

Along those lines, our integrated analysis argues towards a distinctive metabolic outcome, which is a deregulation of the enzymatic route citrulline-arginine-ornithine. Although it would be interesting to decipher if the particular metabolic phenotype relates to alterations in NO levels or in polyamines, this is beyond the scope of the current paper which aims mainly at a systemic description of minimal residual disease.

Lastly, while a deregulation of certain enzymes of the urea cycle and enhanced glycolysis is detected in aggressive breast cancers, the finding that these characteristics remain in residual cells, which are non-proliferative and phenotypically similar to their normal counterparts, is a novel one. In fact, these findings are at the heart of the “metabolic memory” observation, which in turn offers interference possibilities with MRD. We have not encountered such data elsewhere and consider it important - as a concept and as potential targets for long term care.

Minor comments-
S5- hard to compare since they show different metabolites for the intra and extracellular fractions.

→ Intra- and extra-cellular fractions are distinct in their metabolic content, which is consistent with the fact that not all metabolites are secreted / are freely diffusing across the cell membrane.

Fig. 4b- unclear what is being demonstrated. Are these representative images for 4a? The data regarding the metabolic effects on the epigenome are only speculative.

→ We rephrased these sentences (lines 182-187) to make clear that we show representative images (Fig. 4b) for the inhibition assay (Fig. 4a) and also to provide more descriptive detail on the shown phenotypes. We did not mention any metabolic effects on the epigenome in the context of this experiment.

However, the findings on the DNA methylation profiles of residual and tumor cells were investigated further and we now present data on a footprinting based activity analysis of transcription factor target gene expression (including a new Figure S12a-c and Table S3). This new section (lines 201-225) “suggests that the accumulation of certain metabolites affords an additional survival advantage for MRD that is sustained through epigenetic imprinting.”

Referee #2 (Remarks for Author):
Remarks to the Author:

In this manuscript, the authors performed the metabolomics, lipidomics and transcriptomics analysis of minimal residual disease (MRD) based on a tractable organoid system, and uncovered the elevated glycolysis and dysregulated urea cycle in residual organoids, which was also observed in a mouse model and in transcriptomic data from patients upon neo-adjuvant therapy. They further found a similarity in DNA methylation profiles between tumor and residual cells. Intervening glycolysis with a small molecule inhibitor could suppress the cell survival, suggesting a potential target for preventing breast tumor recurrence. This study offers the multi-omics data to study the metabolic alterations of MRD, however, there are a few issues to be clarified.

1. The authors claimed the residual organoids they established were referred to as "residual cells" which constitute the treatment-tolerant cancer cells. However, they did not confirm whether the residual organoids were treatment-tolerant or treatment-resistant. It would be necessary to establish residual organoids with treatment of high dose anti-cancer drugs and determine the metabolic alterations of the surviving residual cells upon drug treatment by multi-omics analysis.

→ We thank the reviewer for pointing to the fact that we did not introduce the system used sufficiently as well as the main outcomes from prior work. We have now provided more context to the cited prior work and rephrased the introduction accordingly (lines 38-48).

In short, a detailed characterization of the residual cells is referenced and can be found in
Jechlinger M, Podsypanina K, Varmus H (2009) Regulation of transgenes in three-dimensional cultures of primary mouse mammary cells demonstrates oncogene dependence and identifies cells that survive deinduction. *Genes & development* 23: 1677-88
And
Havas KM, Milchevskaya V, Radic K, Alladin A, Kafkia E, Garcia M, Stolte J, Klaus B, Rotmensz N, Gibson TJ, Burwinkel B, Schneeweiss A, Pruneri G, Patil KR, Sotillo R, Jechlinger M (2017) Metabolic shifts in residual breast cancer drive tumor recurrence. *J Clin Invest* 127: 2091-2105

We have prior described (i) the dormant nature of the treatment resistant (former) tumor cells that re-acquire a polarized epithelial morphology upon silencing the oncogenes, (ii) the same phenotype, when tumors were interfered with targeted drug treatment instead, (iii) a distinct transcriptomic profile for those residual cells that gave rise to recurrence *in vitro* and *in vivo* following a dormancy period and (iv) had correlated first outcomes obtained with the described system with patient disease.

We believe that this provides enough peer-reviewed evidence to employ the established systems and methods to the current study on a three-way comparison of normal, tumor and residual disease states.

2. In figure 1b and figure S2, the distinct transcriptomics profiling between residual cells and normal cells was not surprising. It is also important to perform the gene-set enrichment analysis comparing the residual and tumor cells to identify the difference between residual cells and tumor cells.

→ We agree with the reviewer that the distinct trancriptomic profiles between residual and normal cells were expected following our publication on this (Havas et al., 2017). However, the closeness of residual cells to the overall metabolism (transcriptomic, metabolomic and epigenetic) of the tumor cells constitutes the -novel- main base for this manuscript.

Previous studies that had examined differences between tumor and MRD lacked a comparison to normal, healthy tissue and -hence- could not identify common de-regulated features of the two disease states as compared to normal tissue. Therefore, a holistic picture of the global molecular characteristics of residual cells (MRD) has thus far been elusive. In other words, a simple comparison of tumor and residual state would not yield a meaningful picture of the MRD, due to their (discovered and shown) similarity. We have integrated these points in the discussion (lines 237-242)

On a conceptual and clinical point of view, we are actually interested in common targets that would be present in both malignant states -residual and tumor- and their inhibition would not impinge on normal tissue/cells.

3. Like the issues in transcriptomics analysis, the authors should analyze the difference between residual cells and tumor cells based on the metabolomic and lipidomic data. This will contribute to identify new biomarkers and targeted therapeutic strategy for MRD.

→ The goal of the present study is to gain system-level insight into metabolic operation and regulation in the residual cells. Biomarker discovery would require a completely different study design that is clinically oriented and is thus beyond the scope of the present molecular study.

4. The authors performed lipidomic analysis but they did not mention the specific alterations in lipids in the manuscript and figures. A more detailed description of the lipidomic profiling would be needed.

→ The goal of the present study is to gain system-level insight and thus a detailed analysis of lipidomics is outside the scope. Lipidomics data cannot be integrated into genome-scale metabolic models at the current state of art. The value of lipidomics data in our case is to provide an orthogonal support to the distinctions between normal, tumor and residual cells as observed at the transcriptomic and metabolomics level.

5. The glycolysis inhibitor 3-BP has been regarded as a potential anti-cancer drug. In figure 4a, the tumor cells stayed close to the baseline viability after exposure to 3-BP is not rational. In fact, we can observe a marked difference in morphology of the tumor cells with or without treatment of 3-BP in figure 4b.

→ 3-BP as an anti-cancer drug and the effect of treatment is hotly debated (as an example, please refer to https://blogs.sciencemag.org/pipeline/archives/2016/08/17/3-bromopyruvate-what-a-mess). In fact, we can see no large treatment effect in our assays [96 well plate assay + on the morphology, Fig.4 and renewed description on morphology in the main text lines 182-186 even at the highest applied doses. These observations are very much in line with reported clinical-trial data.

Regarding the difference in treatment effect of tumor cells and residual cells: We now further extended and renewed the paragraph discussing the potential roots of higher glycolytic dependence of residual cells as compared to tumor cells (line 166-175). We cite and present data towards (1) lower OXPHOS capacity, (2) less anti-apoptotic signal pathway acitivity and (3) reduced metabolic flexibility in residual cells in comparison to tumor cells.

6. To clarity the dynamic metabolism changes, the authors would be better to perform the metabolic flux assessed by stable isotope labeling.

→ We have performed this experiment now (Figure 4d). The new data shows, in agreement with the integrative modelling, that the higher glucose metabolism to lactate in the residual population, as compared to normal cells, is further validated by the increased labeling of lactate following supplementation with [U-13C]glucose.

Referee #3 (Comments on Novelty/Model System for Author):

In this manuscript, a novel concept of metabolic memory is proposed. The authors show that treatment-resistant cancer cells, despite having a quiescent phenotype, still have a metabolic profile similar to cancer cells. They claim that this specific metabolic features represent a vulnerability of the residual cells, notably to glucose deprivation.
This is very original data that could represent, if properly revised, an important finding in the field of cancer metabolism. In the present form, however, the data presented is not yet conclusive enough, and further experiments should be performed.

1. As a result of switching-on Myc expression, upon doxycycline treatment, cells were transformed and generated a tumor-like structure. Myc-expressing cells had a distinct gene expression signature, which was consistent with tumor formation. After the extinction of Myc expression, tumor cells regressed, but the gene expression profile (at least at a significant extent) was similar to the tumor cells. The question here is how the expression of these genes is still changed if the triggering factor, Myc, is absent? Which factors contribute to the sustained expression of these genes?

→ We agree with the referee that the question regarding the nature ot the metabolic memory following silencing of the oncogenes is of utmost relevance. Following the identification of the phenomenon of "metabolic memory" we now highlight transcription factors enriched in the residual cell population (Table S3) and followed the findings of strong Hif1a de-methylation – in sync with the tumor population (Figure S12 and S13).
The observed closeness of the DNA methylation profiles of tumor and residual cells suggests the imprinting of the tumor state in the residual situation (Figure 4e)

2. Lipids composition is shown to be different in tumor cells and in resistant cells, but these differences are not detailed, neither is explained the significance of the distinct lipid profiles.

→ The goal of the present study is to gain system-level insight and thus a detailed analysis of lipidomics is outside the scope. Lipidomics data cannot be integrated into genome-scale metabolic models at the current state of art. The value of lipidomics data in our case is to provide an orthogonal support to the distinctions between normal, tumor and residual cells as observed at the transcriptomic and metabolomics level.

3. Metabolic studies in the cells using Seahorse analysis could provide functional data to support the changes observed in metabolomics and lipidomics studies.

→ We have now included even more direct evidence for flux changes – one based on isotope labelling (Figure 4d). The new data shows, in agreement with the integrative modelling, that the higher glucose metabolism to lactate in the residual population, as compared to normal cells, is further validated by the increased labeling of lactate following supplementation with [U-13C]glucose.

4. In the figure 3, the metabolic profile of residual cancer cells from tumors in mice is compared to normal cells, and it is shown that glycolysis and urea cycle pathways are increased in these cells. However, an important control, tumor cells, is missing in this experiment.

→ We deliberately took the 2 phenotypically similar states (normal and residual, both re-polarized and low in proliferation of cells) for these experiments, since the differences in the tumor population would have made the comparison very complicated, especially regarding size and histology.
The main aim of these experiments was to validate the *in vitro* findings in an *in vivo* like setting, functional assays and interferences with the 3 cell states could only be done in the 3D cell culture settings.

5. The authors compare the transcriptomics data of patients before and after neoadjuvant therapy and show a similar gene expression profile in residual cells. However, under the view of this reviewer, this comparison is not conclusive, because the authors cannot discriminate between residual cells and "remaining" cancer cells. Indeed, they should show that, similar as in the organoids results, the patient samples that they are analyzing also have deregulated cell cycle and cell proliferation markers as well as upregulated cytoskeletal and adhesion genes.

→ We understand the suggestion and rely on the fact that most of these residual tissues have been obtained after substantial time of dormancy, which is a frequent event in breast cancer that sees recurrencies after up to 20 years following successful therapy. We used data from published studies to perform the meta-analysis on the residual cell and tumor cell signatures. The respective controls on the residual state was performed in the original studies and the histology is not available to us.

6.It is also shown that inhibition of glycolysis specifically killed residual cells. This treatment was not efficient, however, to kill tumor cells. This result is counterintuitive, since tumor cells have also increased glycolysis. How tumor cells overcome the inhibition of glycolysis? What alternative pathways are triggered in these cells, which are not active in the residual cells? A comparative analysis would provide some clues about the metabolic changes in residual cells that renders them more sensitive to glucose deprivation.

→ While the glycolytic phenotype was observed for both tumor and residual cells, only the residual cells showed vulnerability to the glycolysis inhibition. While this observation might appear as counterintuitive, it is a direct consequence of the metabolic memory. While the metabolic plasticity of the tumor state allows them to utilize alternative pathways, the residual cells become vulnerable due to their reliance on glycolysis in contrast to the starting normal state. The flux through oxidative phosphorylation (OXPHOS) in relation to the glycolysis was predicted by the model to be lower in both residual and tumor cells than in the normal cells. Yet, transcript levels of OXPHOS associated genes indicated that the tumor cells harbored higher capacity for OXPHOS than utilized at the flux level (Figure S8). The excess capacity in central metabolic pathways has been noted before as a buffer in the face of perturbations (e.g., Park et al., Nat Chem Biol, 2019).

We now further extended and renewed the paragraph discussing the potential roots of higher glycolytic dependence of residual cells as compared to tumor cells (line 167-176). We cite and present data towards (1) lower OXPHOS capacity, (2) less anti-apoptotic signal pathway activity and (3) reduced metabolic flexibility in residual cells in comparison to tumor cells.

Lee JS, Adler L, Karathia H, Carmel N, Rabinovich S, Auslander N, Keshet R, Stettner N, Silberman A, Agemy L, Helbling D, Eilam R, Sun Q, Brandis A, Malitsky S, Itkin M, Weiss H, Pinto S, Kalaora S, Levy R et al. (2018) Urea Cycle Dysregulation Generates Clinically Relevant Genomic and Biochemical Signatures. *Cell* 174: 1559-1570 e22

1st Revision - Editorial Decision 13th Aug 2021

Thank you again for submitting your work to Molecular Systems Biology. We have now heard back from the reviewer who was asked to evaluate your study. Reviewer #4 is a new reviewer, who was given access to the revised manuscript and your responses to the reviewers' comments from EMBO Molecular Medicine. They were asked to evaluate whether the reviewers' concerns have been adequately addressed, and to assess the suitability of the study for publication keeping in mind the editorial criteria and scope of Molecular Systems Biology. As you will see below, the reviewer thinks that the previous concerns have been addressed and is supportive of publication.

Before we formally accept the study for publication, we would ask you to address some editorial issues listed below.

REFEREE REPORTS

Reviewer #4:

This study investigates treatment resistant breast cancer using multi omics approaches with an emphasis on the metabolic network. There is substantial novelty in that quantitative modeling and multi-omics approaches are not regularly used in this area. The paper has been through revisions and the authors have made some substantial effort to improve it. Technical concerns have been addressed. This will be an important study.

2nd Authors' Response to Reviewers 23rd Sep 2021

The authors have made all requested editorial changes.

Thank you again for sending us your revised manuscript and for performing the last requested editorial changes. We are now satisfied with the modifications made and I am pleased to inform you that your paper has been accepted for publication.

EMBO PRESS

YOU MUST COMPLETE ALL CELLS WITH A PINK BACKGROUND ↓

PLEASE NOTE THAT THIS CHECKLIST WILL BE PUBLISHED ALONGSIDE YOUR PAPER

Corresponding Author Name: Kiran R. Patil and Martin Jechlinger

Journal Submitted to: Molecular Systems Biology
Manuscript Number: MSB-2020-10141R

Reporting Checklist For Life Sciences Articles (Rev. June 2017)

This checklist is used to ensure good reporting standards and to improve the reproducibility of published results. These guidelines are consistent with the Principles and Guidelines for Reporting Preclinical Research issued by the NIH in 2014. Please follow the journal's authorship guidelines in preparing your manuscript.

A- Figures

1. Data

The data shown in figures should satisfy the following conditions:

- the data were obtained and processed according to the field's best practice and are presented to reflect the results of the experiments in an accurate and unbiased manner.
- figure panels include only data points, measurements or observations that can be compared to each other in a scientifically meaningful way.
- graphs include clearly labeled error bars for independent experiments and sample sizes. Unless justified, error bars should not be shown for technical replicates.
- if n< 5, the individual data points from each experiment should be plotted and any statistical test employed should be justified
- Source Data should be included to report the data underlying graphs. Please follow the guidelines set out in the author ship guidelines on Data Presentation.

2. Captions

Each figure caption should contain the following information, for each panel where they are relevant:

- a specification of the experimental system investigated (eg cell line, species name).
- the assay(s) and method(s) used to carry out the reported observations and measurements
- an explicit mention of the biological and chemical entity(ies) that are being measured.
- an explicit mention of the biological and chemical entity(ies) that are altered/varied/perturbed in a controlled manner.

- the exact sample size (n) for each experimental group/condition, given as a number, not a range;
- a description of the sample collection allowing the reader to understand whether the samples represent technical or biological replicates (including how many animals, litters, cultures, etc.).
- a statement of how many times the experiment shown was independently replicated in the laboratory.
- definitions of statistical methods and measures:
  - common tests, such as t-test (please specify whether paired vs. unpaired), simple χ2 tests, Wilcoxon and Mann-Whitney tests, can be unambiguously identified by name only, but more complex techniques should be described in the methods section;
  - are tests one-sided or two-sided?
  - are there adjustments for multiple comparisons?
  - exact statistical test results, e.g., P values = x but not P values < x;
  - definition of 'center values' as median or average;
  - definition of error bars as s.d. or s.e.m.

Any descriptions too long for the figure legend should be included in the methods section and/or with the source data.

In the pink boxes below, please ensure that the answers to the following questions are reported in the manuscript itself. Every question should be answered. If the question is not relevant to your research, please write NA (non applicable). We encourage you to include a specific subsection in the methods section for statistics, reagents, animal models and human subjects.

USEFUL LINKS FOR COMPLETING THIS FORM

http://www.antibodypedia.com
http://1degreebio.org
http://www.equator-network.org/reporting-guidelines/improving-bioscience-research-repor
http://grants.nih.gov/grants/olaw/olaw.htm
http://www.mrc.ac.uk/Ourresearch/Ethicsresearchguidance/Useofanimals/index.htm
http://ClinicalTrials.gov
http://www.consort-statement.org
http://www.consort-statement.org/checklists/view/32-consort/66-title
http://www.equator-network.org/reporting-guidelines/reporting-recommendations-for-tumo
http://datadryad.org
http://figshare.com
http://www.ncbi.nlm.nih.gov/gap
http://www.ebi.ac.uk/ega
http://biomodels.net/
http://biomodels.net/miriam/
http://jjj.biochem.sun.ac.za
http://oba.od.nih.gov/biosecurity/biosecurity_documents.html
http://www.selectagents.gov/

B- Statistics and general methods

Please fill out these boxes ↓ (Do not worry if you cannot see all your text once you press return)

1.a. How was the sample size chosen to ensure adequate power to detect a pre-specified effect size?	All experiments were performed using biological and technical replicates. No prior assumptions were made regarding effect sizes, and thus no calculations for sample size were performed a priori. A full description of the statistical parameters can be found in figure legends and Methods section.
1.b. For animal studies, include a statement about sample size estimate even if no statistical methods were used.	No prior assumptions were made regarding effect sizes. Sample sizes were chosen based on the requirement for statistical tests and feasibility. All relevant and possible data points were used for statistical comparisons when applicable.
2. Describe inclusion/exclusion criteria if samples or animals were excluded from the analysis. Were the criteria pre-established?	No samples were excluded from further analysis.
3. Were any steps taken to minimize the effects of subjective bias when allocating animals/samples to treatment (e.g. randomization procedure)? If yes, please describe.	For in vitro experiments in 3D culture, assignmnet to experimental group was done at random post orgaoid culturing. In short: cells were seeded in equal amounts and under the same conditions; structures undergoing tumor induction and regression were compared to the not-induced (normal, control) state, collected at the same timepoint of the experiment.
For animal studies, include a statement about randomization even if no randomization was used.	For experiments in vivo, animals were allocated into experimental groups by their genotype (i.e. inducible, uninducible). The potential confounding effect of doxycycline used for tumor induction was tested by treating uninducible animal samples. Within the animals of a given genotype, experimental groups were assigned at random.
4.a. Were any steps taken to minimize the effects of subjective bias during group allocation or/and when assessing results (e.g. blinding of the investigator)? If yes please describe.	Blinding was done for immunofluorescence staining for ARG1: tissue section samples from two different groups (age-matched control-healthy animals, animals with regressed tumors) were coded.
4.b. For animal studies, include a statement about blinding even if no blinding was done	Regarding animal groups, blinding was not possible, due to the need to maintain specific experimental conditions for tumor induction/regression.
5. For every figure, are statistical tests justified as appropriate?	Yes
Do the data meet the assumptions of the tests (e.g., normal distribution)? Describe any methods used to assess it.	Yes, described in materials and methods.
Is there an estimate of variation within each group of data?	Yes, described in figures and can be estimated from the Source / raw data.

Is the variance similar between the groups that are being statistically compared?	Case-dependent. Data variance shown in Figures / available in the source data.

C- Reagents

6. To show that antibodies were profiled for use in the system under study (assay and species), provide a citation, catalog number and/or clone number, supplementary information or reference to an antibody validation profile. e.g., Antibodypedia (see link list at top right), 1DegreeBio (see link list at top right).	All antibodies used are listed in the Materials and Methods section submitted (Radic et al., sections Immunofluorescence and Immunohistochemistry); We state in detail provider, catalog number, dilution used; Information for species, application and suggested dilutions were taken from respective manufactors' instructions and citated manuscripts therein. Antibodies were titrated and controls involved background evaluation omitting the secondary antibody.
7. Identify the source of cell lines and report if they were recently authenticated (e.g., by STR profiling) and tested for mycoplasma contamination.	NA

* for all hyperlinks, please see the table at the top right of the document

D- Animal Models

8. Report species, strain, gender, age of animals and genetic modification status where applicable. Please detail housing and husbandry conditions and the source of animals.	Mus musculus; strains backcrossed into FVB background; virgin females; 8-10 weeks old at the start of the experiments; TetO-cMYC/TetO-Neu/MMTV-rtTA<br>Animals were kept on a 12-hour light/12-hour dark cycle with a constant ambient temperature (23±1°C) and humidity (60±8%), supplied with food pellets (for tumor induction the pellets contained doxycycline hyclate, 625 mg/kg; Envigo Teklad) and water ad libitum.
9. For experiments involving live vertebrates, include a statement of compliance with ethical regulations and identify the committee(s) approving the experiments.	Breeding and maintenance of the mouse colony was done in accordance to the guidelines of the European Commission, revised Directive 2010/63/EU and AVMA Guidelines 2007, under veterinarian supervision. The principles of 3Rs were followed in this study.
10. We recommend consulting the ARRIVE guidelines (see link list at top right) (PLoS Biol. 8(6), e1000412, 2010) to ensure that other relevant aspects of animal studies are adequately reported. See author guidelines, under 'Reporting Guidelines'. See also: NIH (see link list at top right) and MRC (see link list at top right) recommendations. Please confirm compliance.	we confirm compliance

E- Human Subjects

11. Identify the committee(s) approving the study protocol.	NA
12. Include a statement confirming that informed consent was obtained from all subjects and that the experiments conformed to the principles set out in the WMA Declaration of Helsinki and the Department of Health and Human Services Belmont Report.	NA
13. For publication of patient photos, include a statement confirming that consent to publish was obtained.	NA
14. Report any restrictions on the availability (and/or on the use) of human data or samples.	NA
15. Report the clinical trial registration number (at ClinicalTrials.gov or equivalent), where applicable.	NA
16. For phase II and III randomized controlled trials, please refer to the CONSORT flow diagram (see link list at top right) and submit the CONSORT checklist (see link list at top right) with your submission. See author guidelines, under 'Reporting Guidelines'. Please confirm you have submitted this list.	NA
17. For tumor marker prognostic studies, we recommend that you follow the REMARK reporting guidelines (see link list at top right). See author guidelines, under 'Reporting Guidelines'. Please confirm you have followed these guidelines.	NA

F- Data Accessibility

18: Provide a "Data Availability" section at the end of the Materials & Methods, listing the accession codes for data generated in this study and deposited in a public database (e.g. RNA-Seq data: Gene Expression Omnibus GSE39462, Proteomics data: PRIDE PXD000208 etc.) Please refer to our author guidelines for 'Data Deposition'.<br><br>Data deposition in a public repository is mandatory for:<br>a. Protein, DNA and RNA sequences<br>b. Macromolecular structures<br>c. Crystallographic data for small molecules<br>d. Functional genomics data<br>e. Proteomics and molecular interactions	The datasets obtained in this study are available in the following repositories: RNAseq data are available at ArrayExpress database at EMBL-EBI (www.ebi.ac.uk/arrayexpress) under accession number E-MTAB-8834. Enzymatic Methyl-seq (EM-seq) data are submitted to ArrayExpress and currently are available on request. GC-MS targeted metabolomics data are submitted to MetaboLights database (https://www.ebi.ac.uk/metabolights/MTBLS1507). GC-MS targeted metabolomics data, FIA-MS untargeted metabolomics data and Lipidomics data are available at Mendeley (https://data.mendeley.com/datasets/8gby9dxh83/draft?a=fb9d4bd8-0acd-47d9-bb27-607b56e89d63).
19. Deposition is strongly recommended for any datasets that are central and integral to the study; please consider the journal's data policy. If no structured public repository exists for a given data type, we encourage the provision of datasets in the manuscript as a Supplementary Document (see author guidelines under 'Expanded View' or in unstructured repositories such as Dryad (see link list at top right) or Figshare (see link list at top right).	NA
20. Access to human clinical and genomic datasets should be provided with as few restrictions as possible while respecting ethical obligations to the patients and relevant medical and legal issues. If practically possible and compatible with the individual consent agreement used in the study, such data should be deposited in one of the major public access-controlled repositories such as dbGAP (see link list at top right) or EGA (see link list at top right).	NA
21. Computational models that are central and integral to a study should be shared without restrictions and provided in a machine-readable form. The relevant accession numbers or links should be provided. When possible, standardized format (SBML, CellML) should be used instead of scripts (e.g. MATLAB). Authors are strongly encouraged to follow the MIRIAM guidelines (see link list at top right) and deposit their model in a public database such as Biomodels (see link list at top right) or JWS Online (see link list at top right). If computer source code is provided with the paper, it should be deposited in a public repository or included in supplementary information.	The human genome-scale metabolic model HMR2 revised during the current study and used for simulation is available at: https://github.com/katharinazirngibl/MetabolicModeling-MinimalResidualDesease

G- Dual use research of concern

22. Could your study fall under dual use research restrictions? Please check biosecurity documents (see link list at top right) and list of select agents and toxins (APHIS/CDC) (see link list at top right). According to our biosecurity guidelines, provide a statement only if it could.	NA
